# Effects of different fertilization practices on maize yield, soil nutrients, soil moisture, and water use efficiency in northern China based on a meta-analysis

**DOI:** 10.1038/s41598-024-57031-z

**Published:** 2024-03-18

**Authors:** Minghao Jiang, Chao Dong, Wenpeng Bian, Wenbei Zhang, Yong Wang

**Affiliations:** 1 School of Water Conservancy and Civil Engineering, Northeast Agricultural University, Harbin, 150030 China; 2 Inner Mongolia University of Technology, Hohhot, 010051 Inner Mongolia China; 3 College of Horticulture and Landscape Architecture, Northeast Agricultural University, Harbin, 150030 China

**Keywords:** Ecology, Environmental sciences

## Abstract

The application of fertilizer to ensure the steady improvement of crop yield has become the main means of agricultural production. However, it remains to be determined whether fertilization practices with different combinations of nitrogen (N), phosphorus (P), potassium (K), and organic (O) fertilizers play a positive role in the sustainability of maize yield and the soil in which it is grown. Therefore,this meta-analysis extracted 2663 data points from 76 studies to systematically analyze and explore the effects of different fertilization measures on maize yield, soil nutrients, water content and water use efficiency (WUE) in northern China. Articles addressing this topic showed that fertilization effectively increased the soil nutrient content and maize yield. The soil organic matter (SOM) increased by 2.36 (N)–55.38% (NPO), total nitrogen content increased by 6.10 (N)–56.39% (NPO), available phosphorus content increased by 17.12 (N)–474.74% (NPO), and available potassium content changed by − 2.90 (NP)–64.40% (NPO). Soil moisture increased by 3.59% under a single organic fertilizer application and decreased by 4.27–13.40% under the other treatments. Compared with no fertilization, the yield increase of fertilized maize reached 11.65–220.42%. NP, NPK and NPKO contributed the most to increased yield in lithological, black and fluvo-aquic soils, respectively. The effects of different fertilization practices on maize yield varied in response to the same meteorological factors. The WUE increased from 9.51 to 160.72%. In conclusion, rational fertilization can improve the soil nutrient content and increase maize yield. The combined application of chemical and organic fertilizer showed the greatest increase in yield and WUE. Organic fertilizer application alone increased soil moisture. Our results provide a theoretical basis for fertilizer application and for improving the soil structure for maize cultivation in northern China.

## Introduction

Maize is one of the main cereal crops in China. Compared with wheat, rice and other crops, the maize root system can penetrate deeper into the soil in search of water and nutrients, and the leaves have a higher transpiration rate and photosynthetic efficiency^1^. This ensures the more efficient use of light energy for photosynthesis while regulating water loss by opening or closing the stomata, thus increasing the ability to synthesize carbohydrates and the growth rate, making maize more resistant to water stress under drought conditions and better able to adapt to poor soil conditions^2^. Combined with its own environmental adaptations and its long history of domestication, maize has demonstrated excellent environmental adaptability^3^. Since 1950, maize cultivation in China has been growing, and technology has been improving, making China the second-largest maize-producing country in the world^4^. Maize is not only a food product but also a raw material for the development of industry, animal husbandry and other fields in China. Therefore, China is also the second-largest consumer of maize worldwide^5^. According to the statistical data from the National Bureau of Statistics of China, in 2020, the total area planted with maize in China reached 23,056 thousand hectares. The total output reached 192,590.4 kt, with the Northeast Plain and the North China Plain accounting for approximately 70% of the total national maize output^6^. Around the same period, the yield of maize per unit area in six provinces (Heilongjiang, Jilin, Liaoning, Inner Mongolia, Shandong and Jiangsu) exceeded 400 kg per mu in 2019, reaching the highest yield in Jilin Province, 7.2 tons per hectare^7^. Northern China, as the main area of intensive maize cultivation, has contributed greatly to ensuring food security in China and even the world, as well as the stable development of other industries^8^. This has led to the rapid growth of the corn farming industry and raises questions about how long-term corn farming affects soil moisture and nutrients. Is there an indirect effect on yield? Does fertilization play a positive role in this process?

Fertilizer application is an important artificial measure, and the provision of adequate nutrients to crops through fertilization is critical to maintaining high crop yields and ensuring global food security^9^. Fertilizer application is responsible for up to 50% of the total increase in crop yields in modern agricultural production^10^. Fertilizer application ensures that maize absorbs sufficient nutrients from the soil as it grows to sustain its own biomass build-up and the growth of its photosynthetic organs^11,12^. At present, most studies have shown that the application of nitrogen, phosphorus and potassium fertilizers has a significant impact on soil fertility^13,14^. The combined application of organic fertilizer and chemical fertilizers can further improve soil nutrients and the organic matter content^15–17^. However, some studies showed that potassium^18^ and phosphorus^19^ availability in the soil can be reduced by fertilizing the soil with NPK compared to not fertilizing. The combined application of organic fertilizer and chemical fertilizer or chemical fertilizer alone reduced the soil total nitrogen content^20,21^. In addition, fertilization can change soil chemical properties and affect soil moisture^22,23^. Through a 3-year field experiment, Wang et al.^24^ found that soil water storage in the 0–120 cm soil layer was lower in unfertilized soil compared with fertilized soil; however, Zhang et al.^25^ found that the soil water storage capacity in the 0–200 cm soil layer gradually decreased with increasing fertilizer application, and this phenomenon was particularly obvious in deep soil. Jia et al.^26^ believed that the long-term application of inorganic fertilizer would gradually transfer the water loss problem in the surface soil to deeper soil. Guo et al.^27^ showed that the long-term application of organic fertilizer significantly increased soil water storage, and the increase rate was positively correlated with the application amount and time. Zhang et al.^28^ found that combining organic fertilizer with inorganic fertilizer reduced evaporation and increased the soil water content. On the other hand, some studies showed that improvements in maize yield varied regionally. For example, long-term trials have shown that in the arid region of the Loess Plateau of Northwest China, the application of N fertilizer alone increased maize yield by 10–40% compared with the control^29^; and the addition of organic fertilizer increased maize yield by 1–3 times^30^; On the North China Plain, the combination of organic fertilizer and chemical fertilizer increased maize yield by approximately sixfold compared with no fertilizer treatment^31^. The yield in Northeast China was similar to that in Northwest China under the same fertilizer application, but the demand for fertilizer was lower^32,33^. It has also been reported that not applying P fertilizer for more than ten years in fields with a high P content did not affect the maize yield^34,35^.

To date, Chinese researchers have made many advancements in understanding the effects of fertilization on soil nutrients, moisture and maize yield. However, due to the vast size of China and the large differences within each region, complex external factors such as the regional scale, climate and environment can vary among maize planting regions^36,37^. As a result, summarizing these differences among studies is difficult. In addition, we assume that the soil under different fertilizer application does not always tend to have the same change in each index. Meta-analysis is a formal statistical method used to systematically combine the results of independent experiments and can also be used to quantitatively evaluate the effects of certain treatments at the regional level. This approach has the advantage of analyzing and summarizing large-scale ecological phenomena in a regional environment^38,39^. Therefore, based on the literature, we took northern China as the research area and conducted a meta-analysis to explore the effects of different fertilization measures on maize yield, soil nutrients, soil moisture, and WUE in maize fields and to relate soil types, climate and other factors to quantify the effects of fertilization. We also revealed the mechanisms by which fertilization measures maintain maize yield and preserve soil water fertility. The results showed that crop growth was affected not only by multiple factors but also by multiple interactions that need to be further investigated. This study provides a basis for evaluating the effectiveness of fertilization practices in the rational development and sustainable management of maize soil.

## Materials and methods

### Data collection and extraction

In this study, articles were retrieved using data and knowledge content platforms such as China National Knowledge Infrastructure (https://www.cnki.net), Wanfang (https://www.wanfangdata.com.cn), China Science and Technology Journal Database (http://qikan.cqvip.com), Web of Science (https://www.webofscience.com), and Google Scholar (https://scholar.google.com). The keywords “maize/corn”, “fertilization”, “soil nutrients”, “soil moisture content”, “yield”, and “water use efficiency” were used to retrieve articles in English and Chinese. The articles retrieved were published before May 25, 2023 and included maize field experiments conducted in northern China.

To ensure the quality of the included literature and to reduce bias in the meta-analysis results, the abstracts were read in a preliminary screening, and a full-text reading required meeting the following five inclusion criteria: (1) a maize field experiment was conducted in northern China (Heilongjiang, Jilin, Liaoning, Inner Mongolia, Hebei, Beijing, Tianjin, Shandong, Henan, Shanxi, Shaanxi and northern Anhui provinces) stating the number of repeated experiments, excluding pot experiments or laboratory model simulation experiments; (2) at least one pair of fertilization treatments (organic fertilizer, chemical fertilizer, organic fertilizer combined with chemical fertilizer) and a nonfertilization treatment, with other conditions identical, were conducted; (3) the results provided at least one soil nutrient, water content, yield, or WUE index, and the index under different treatments was reported using the same measurement method and form; (4) if the data were reported in the form of a bar chart, line chart or scatter plot, GetData Graph Digitizer software (version 2.20; GetData; http://getdata-graph-digitizer.com/download.php) was used for data extraction; and (5) the results of studies with identical experimental location, year, variety and experimental data were either excluded or pooled, and repeated data were included only once.

In addition, to prevent a high or low application of chemical fertilizers and the diversity of organic fertilizers from impacting the analysis, the present study required N, P, and K at 150–300 kg/hm^2^, 70–200 kg/hm^2^, and 70–200 kg/hm^2^, respectively, and restricted the organic fertilizers to be derived from livestock manure. To further analyze the effect of fertilizer application on the soil water content, the soil depth of 0–200 cm was divided into three profiles in this study: 0–70 cm, 0–100 cm and 100–200 cm.

### Data transformation and inclusion

We extracted the data from the included articles, and as some of the data on the maize yield, WUE and soil nutrients other than the soil organic matter content were provided in the articles, they were used directly in the analysis. The soil moisture and soil organic carbon ($SOC$) data were standardized according to the methods recommended in the literature, and the soil mass moisture content ($w$)^40^ and soil organic matter ($SOM$)^41^ content were obtained. The specific conversion formulas are as follows:

1 $$w=\frac{{M}_{w}}{{M}_{s}}$$

2 $$V=w\times \rho$$

3 $$SHS=\rho \times h\times w$$

4 $$SOM=SOC\times 1.724$$

where $w$ is the soil mass water content, %; ${M}_{w}$ is the soil water quality, g; ${M}_{s}$ is the soil dry weight, g; $V$ is the soil volumetric water content, %; $\rho$ is the soil bulk density, g cm^−3^; $h$ is the soil layer thickness, mm; $SHS$ is the soil water storage, mm; and $SOC$ is the soil organic carbon content, g kg^−1^.

After data transformation, the sample size of each fertilization measure under each index was determined; studies with a sample size less than 5 were abandoned, and no analysis was carried out.

**Figure 1:**
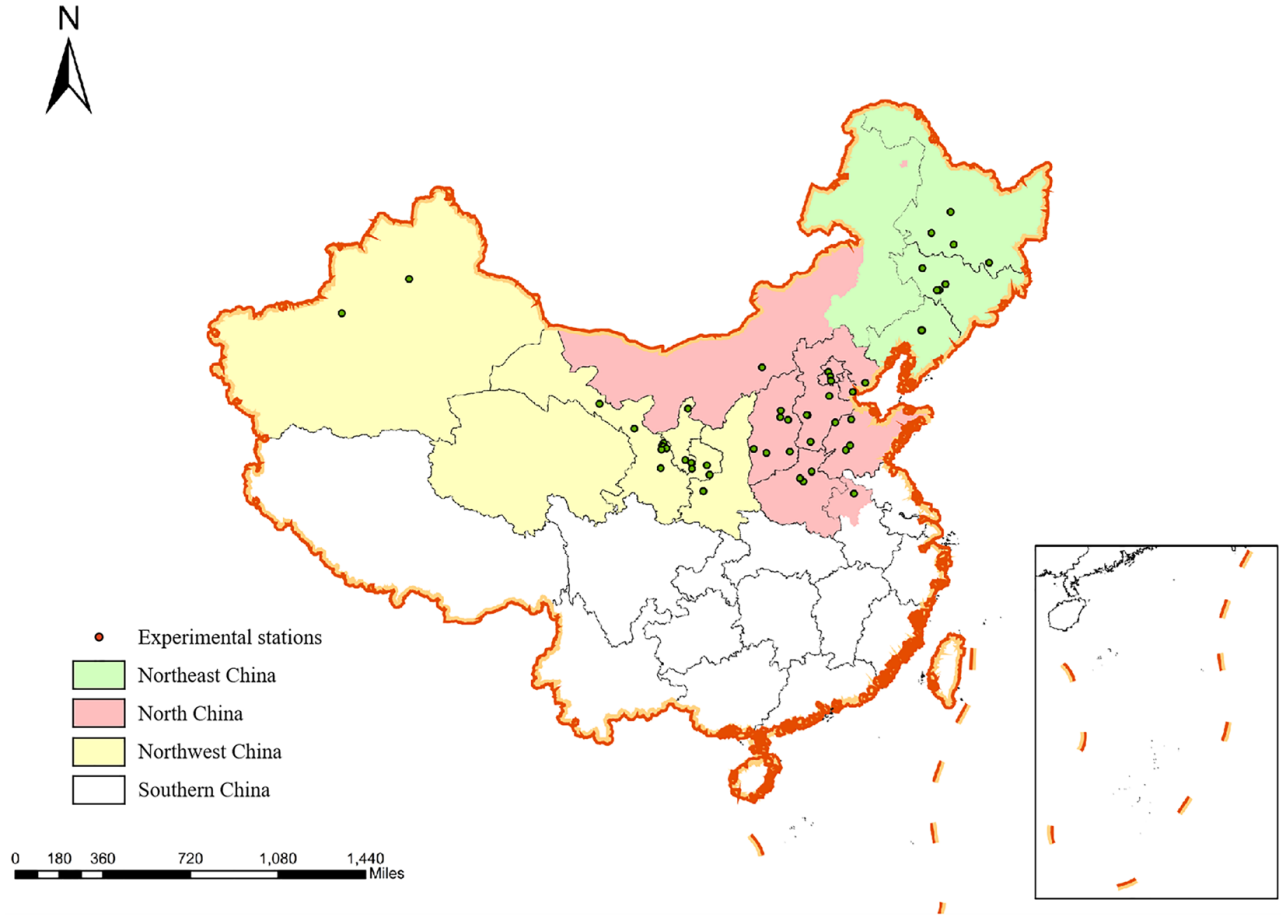
Distribution map of experimental stations (50 test sites). This figure was generated by ArcGIS software (version 10.1; http://support.esrichina-bj.cn/2013/0128/1677.html).

Finally, 76 articles were included, and a total of 2663 pairs of data were analyzed (Supplementary Information). The distribution of experimental sites found in the literature is shown in Fig. 1, the basic information of each experimental site is listed in Table 1 by administrative region, and the sample size for each indicator is shown in Table 2.

**Table 1:** Basic information of the research area described in the paper.

Region	Type of fertilization	Type of soil
Heilongjiang	NP, PK, NPK, O, NPO, NPKO	Black soil
Jilin	N, NP, NK, PK, NPK, O, NO, NPKO	Black soil
Liaoning	NPK, NPKO	Black soil
Inner Mongolia	NP, NK, PK, NPK	Chestnut soil
Beijing	N, NP, NK, PK, NPK, NPKO	Fluvo-aquic soil, cinnamon soil
Tianjin	NP, NK, PK, NPK	Fluvo-aquic soil
Hebei	NP, NK, PK, NPK, NPKO	Fluvo-aquic soil, cinnamon soil, brown soil
Henan	N, NP, NK, NPK, NPKO	Fluvo-aquic soil, brown soil
Anhui	NPK	Fluvo-aquic soil
Shandong	NP, NK, PK, NPK, O, NPKO	Black soil, fluvo-aquic soil, brown soil
Shanxi	N, NP, NPK, O, NO, NPO, NPKO	Cinnamon soil
Gansu	N, NP, NK, PK, NPK, O, NO, NPO, NPKO	Fluvo-aquic soil, cinnamon soil, chestnut soil, lithologic soil, desert soil
Shaanxi	NP, NK, PK, NPK	Fluvo-aquic soil, cinnamon soil
Ningxia	NP, NK, PK, NPK	Lithologic soil
Xinjiang	PK, NPK, NPKO	Desert soil

*N* nitrogen fertilizer, *NP* combined application of nitrogen and phosphate fertilizer, *NK* nitrogen and potassium fertilizer combination, *PK* combined application of phosphate and potassium fertilizer, *NPK* combined application of nitrogen, phosphorus and potassium fertilizer, *O* organic fertilizer, *NO* organic fertilizer with nitrogen fertilizer, *NPO* organic fertilizer with nitrogen and phosphate fertilizer, *NPKO* organic fertilizer with NPK fertilizer.

**Table 2:** Articles including data on maize yield, soil nutrients, water content and water use efficiency.

Indicator	Soil moisture	Soil nutrients				Yield	Water use efficiency
		Organic matter	Total nitrogen	Available phosphorus	Available potassium		
Number of articles	17	18	20	24	24	65	14
Sample Size	1061	137	159	193	193	920	172

### Data analysis and statistics

Meta-analysis is a systematic quantitative analysis and comprehensive evaluation of similar results in the context of multiple independent research results. In essence, it is a series of processes that summarize the results of multiple studies with the same research purpose and analyze and evaluate the comprehensive effect size. It is a statistical method to compare and integrate the conclusions of a series of studies. In this study, fertilizer measures applying any one or more of N, P, K, and O were selected as the treatment group, and no fertilizer measures were selected as the control group to assess the effect of fertilizer measures on maize yield, soil nutrients, soil water content and water use efficiency in terms of natural logarithmic response ratios ($lnR$) using the following equation:

5 $$lnR=ln(\frac{{X}_{t}}{{X}_{c}})=ln{(X}_{t})-ln({X}_{c})$$

where ${X}_{t}$ is the value of the fertilization treatment and ${X}_{c}$ is the value of the no-fertilization treatment.

After the variance ${v}_{i}$ and weight ${w}_{i}$ of the results of each independent study were calculated, the effect size of each study was variance weighted according to this method to obtain the overall weighted comprehensive effect size $lnRR$. The formulas are as follows:

6 $${v}_{i}=\frac{{{SD}_{t}}^{2}}{{N}_{t}\overline{{X }_{t}}}+\frac{{{SD}_{c}}^{2}}{{N}_{c}\overline{{X }_{c}}}$$

7 $${w}_{i}=\frac{1}{{v}_{i}}$$

8 $$lnRR=\frac{\sum (lnRR\times {w}_{i})}{\sum {w}_{i}}$$

where ${{\text{SD}}}_{{\text{t}}}$ and ${{\text{SD}}}_{{\text{c}}}$ are the standard deviations of the treatment group and the control group, respectively. ${{\text{N}}}_{{\text{t}}}$ and ${{\text{N}}}_{{\text{c}}}$ are the sample sizes of the treatment and control groups, respectively, and $\overline{{X }_{t}}$ and $\overline{{X }_{c}}$ are the means of the treatment and control groups, respectively.

Additionally, in the absence of the standard deviation and standard error based on the data collected in this study, the bootstrap method was used to estimate the 95% confidence interval (95% CI) of the statistic^37^. When the sample size was small, the bias correction method was used to correct the 95% CI to obtain the upper limit ($UL$) and lower limit ($LL$) of the corrected confidence interval, calculated as follows:

9 $$UL=N\left[2{N}^{-1}(F)+{Z}_{a/2}\right]$$

10 $$LL=N\left[2{N}^{-1}(F)-{Z}_{a/2}\right]$$

where ${\text{N}}$ is the standard normal distribution function; ${{\text{N}}}^{-1}$ is the inverse function of the standard normal distribution function; ${\text{F}}$ is the ratio of the number of bootstrap values below the initial value to the total number of bootstrap values; ${{\text{Z}}}_{{\text{a}}/2}$ is the ${\text{Z}}$ value of the standard normal distribution, and a is the level of significance, which is taken to be 0.05, so that ${{\text{Z}}}_{{\text{a}}/2}$ is 1.96.

To facilitate observing the effect of the fertilization treatment compared with the control group, the weighted average effect size $lnRR$ was converted to the relative rate of change ($E$) as described, and the calculation formula is as follows:

11 $$E=({e}^{lnRR}-1)\times 100\%$$

The heterogeneity test, also known as the homogeneity test, was used to determine the analysis model. If the test result was not significant (I^2^ < 50%), the fixed effect model was selected, and if I^2^ > 50%, the random effect model was selected. If the confidence interval included zero points, there was no significant difference (P > 0.05); otherwise, there was a significant difference between the treatment group and the control group (P < 0.05). If the confidence interval was greater (or less) than zero, the current fertilization conditions improved (or reduced) the maize yield, soil nutrients, soil water content and WUE.

## Results

**Figure 2:**
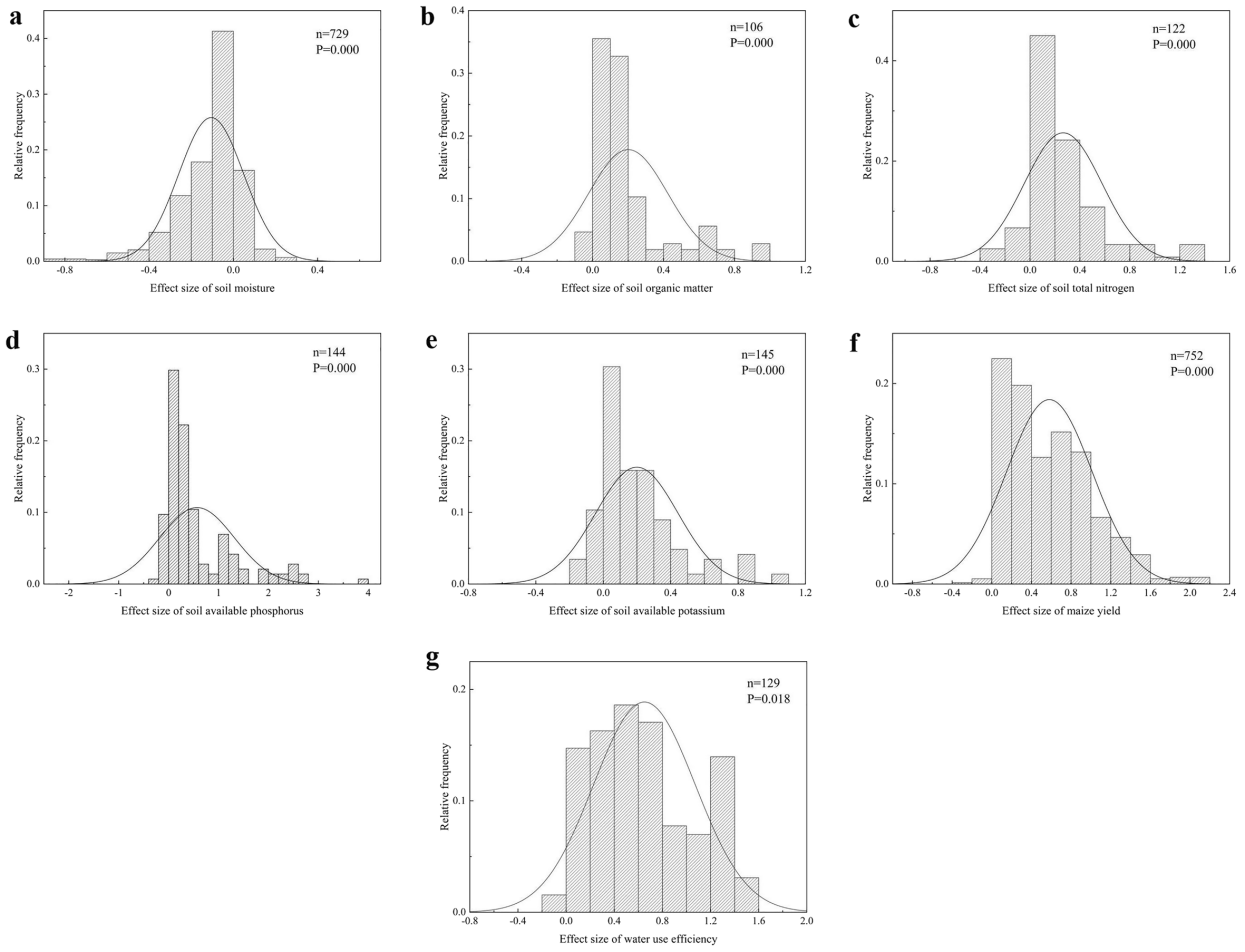
Frequency distribution of maize yield, soil nutrients, water content and water use efficiency under the influence of fertilization. (**a**) Soil moisture. (**b**) Soil organic matter. (**c**) Total nitrogen. (**d**) Available phosphorus. (**e**) Available potassium. (**f**) Maize yield. (**g**) Water use efficiency.

Test of heterogeneity and bias for determining the effect of fertilization on the maize yield, soil nutrients, water content and WUE. Figure 2 shows the distribution of the different effects of fertilization on the maize yield, soil nutrients, water content, and WUE. The results of the Kolmogorov–Smirnov (K-S) test showed that the frequency distribution of the effect sizes related to the maize yield, soil nutrients, water content, and WUE under different fertilization treatments did not follow a normal distribution. Therefore, a nonparametric estimation method was used to generate a comprehensive effect size ($lnRR$), and the bootstrap method was used to estimate the 95% CI of the statistic.

### Response of soil moisture to fertilization in maize fields

**Figure 3:**
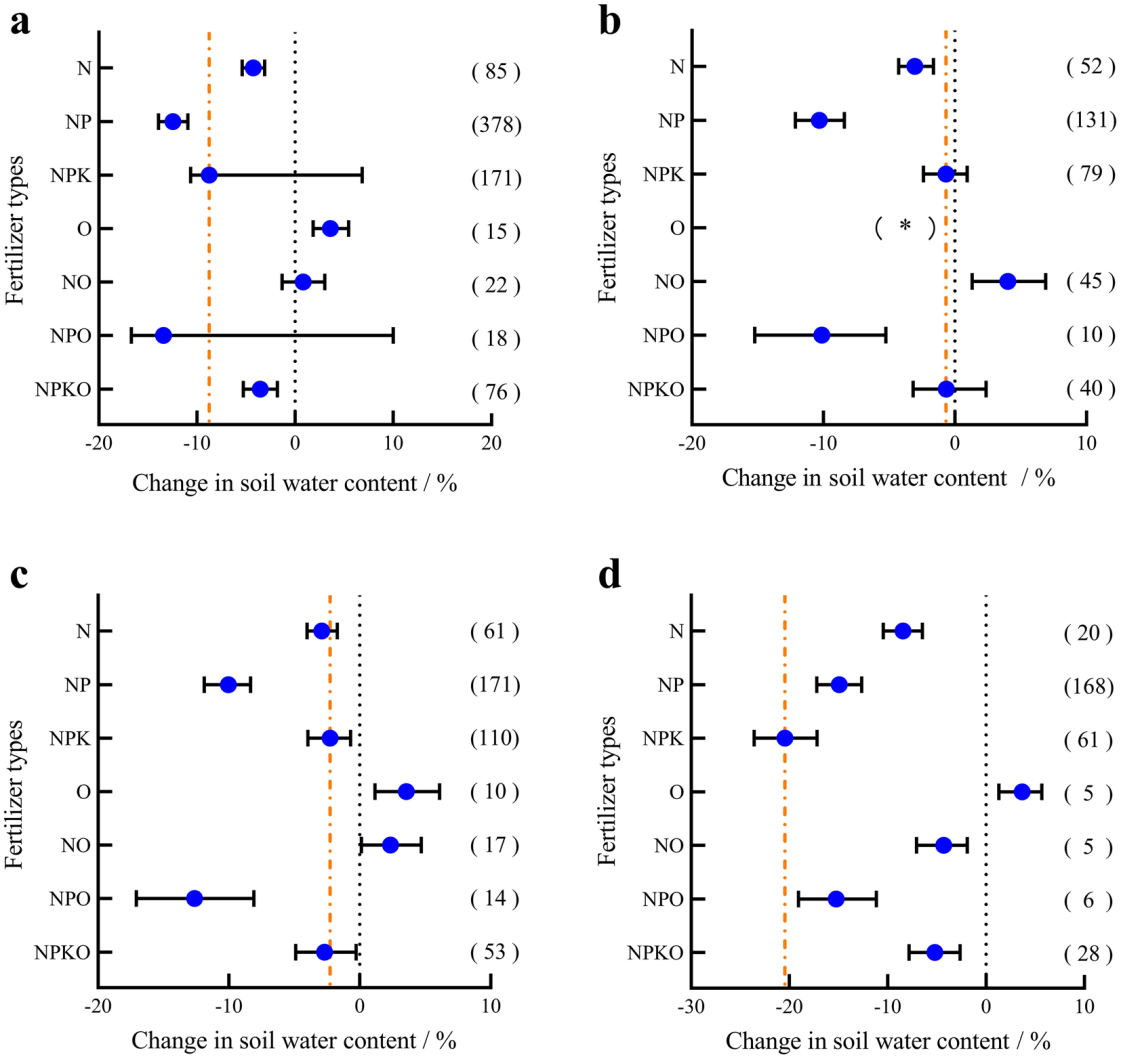
Response of soil moisture in maize fields to different fertilization measures. (**a**) 0–200 cm soil profile. (**b**) 0–70 cm soil profile. (**c**) 0–100 cm soil profile. (**d**) 100–200 cm soil profile. "(*)" indicates that the sample under this treatment was not included in the study due to its small size (n < 5).

Compared with the treatment without fertilization, the effects of fertilization on the water content in the 0–200 cm soil layer (Fig. 3a) were not homogeneous. Organic fertilizer (O) alone significantly increased the soil water content (3.59%, P < 0.05), while organic fertilizer combined with nitrogen fertilizer (NO) had no significant effect on the soil water content (0.8278%, P > 0.05). Organic fertilizer combined with nitrogen and phosphorus fertilizer (NPO) did not significantly reduce soil moisture (P > 0.05), while other fertilization measures significantly reduced soil moisture, with relative rates of change of -3.56% (NPKO), 4.27% (N), and -12.46% (NP).

At the 0–70 cm depth (Fig. 3b), NPO, NP and N significantly reduced soil moisture (P < 0.05), with relative rates of change of − 10.13%, − 10.32% and − 3.05%, respectively, while NPKO and NPK had no effect on soil moisture (P > 0.05). However, NO significantly increased soil moisture (4.0187, P < 0.05). In the 0–100 cm soil layer (Fig. 3c), the effects of NPKO and NPK on the soil water content changed from no effect to a reduction in the soil water content. However, in the 100–200 cm soil profile (Fig. 3d), except for a significant increase in soil moisture due to O (P < 0.05), other fertilization measures significantly reduced the soil moisture (P < 0.05), and the rates of change from the top of the profile to the bottom were as follows: − 15.26%, − 5.20%, − 20.45%, − 14.93%, − 4.32% and − 8.45%.

### Responses of soil nutrients to fertilization in maize fields

**Figure 4:**
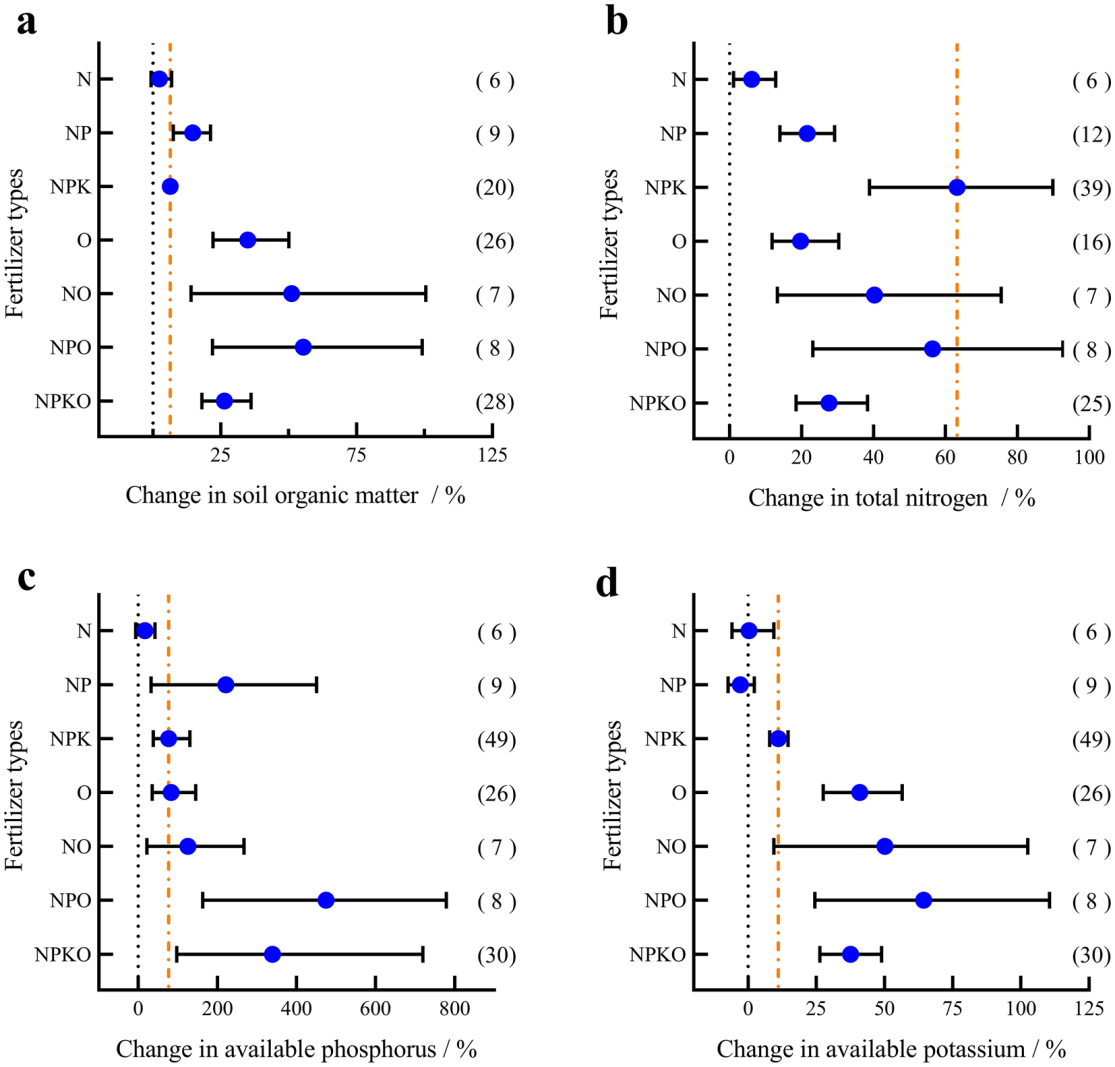
Response of soil nutrients to different fertilization measures in maize fields. (**a**) Soil organic matter. (**b**) Total nitrogen. (**c**) Available phosphorus. (**d**) Available potassium.

Compared to the nonfertilizer treatment, the application of organic fertilizer positively affected soil organic matter (Fig. 4a), with increases of 34.95% (O), 55.38% (NPO), 26.35% (NPKO) and 51.10% (NO), which were higher than the average rate of change with chemical fertilizer application alone [6.31% (NPK), 14.62% (NP), 2.36% (N)]. All fertilization measures increased the soil total nitrogen content (Fig. 4b), and the rate of change from highest change to smallest was as follows: NPK (63.27%) > NPO (56.39%) > NO (40.23%) > NPKO (27.58%) > NP (21.52%) > O (19.77%) > N (6.10%). Regarding the relative rate of change in the soil available P content (Fig. 4c), N resulted in a nonsignificant increase (17.12%, P > 0.05), while other fertilization measures significantly increased available P. Among these, the NPO and NPKO applications showed the most significant increase, increasing soil available P content 4.74-fold and 3.39-fold compared with the control group, with increases ranging from 1.62–7.79 times and 0.96–7.19 times, respectively. The rates of change in the soil available P content under other fertilization treatments from highest to lowest were: NP (221.74%) > NO (125.28%) > O (83.32%) > NPK (76.91%). Except N, which resulted in a nonsignificant reduction in the available potassium content (Fig. 4d) (0.36%, P > 0.05) and NP which did not significantly reduce available potassium content (− 2.90%, P > 0.05), the application of organic fertilizer or potassium fertilizer increased the content of available potassium in the soil compared to no fertilizer. The relative increases in the available potassium content from highest to smallest were NPO (64.40%) > NO (50.11%) > O (40.95%) > NPK (37.55%) > NPK (11.09%).

### Response of maize yield to fertilization

**Figure 5:**
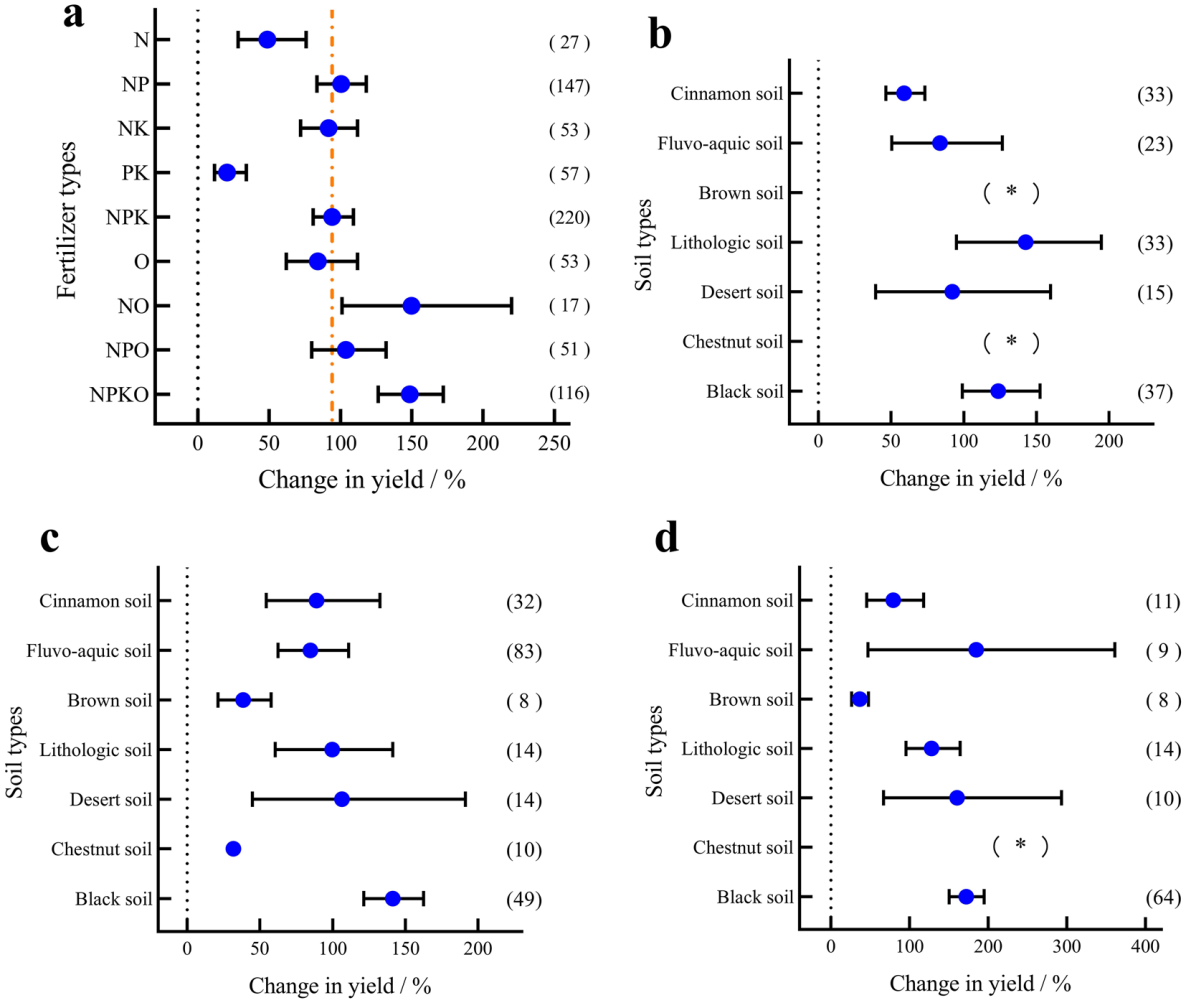
Maize yield and soil type under different fertilization treatments. (**a**) Maize yield under different fertilization treatments. (**b**) Maize yield in different soil types under NP fertilization. (**c**) Maize yield in different soil types under NPK fertilization. (**d**) Maize yield in different soil types under NPKO fertilization.

Compared to the nonfertilizer treatment, fertilization increased maize yield (Fig. 5a) by 11.65–220.42%. The NPKO and NO treatments resulted in the highest maize yield increases of 148.64% and 149.83%, respectively, with the remaining treatments from highest to lowest yield increase being as follows: NPO (103.78%) > NP (100.38%) > NPK (94.11%) > NK (91.71%) > O (84.00%) > N (48.66%) > NK (20.45%). Since NP (Fig. 5b), NPK (Fig. 5c) and NPKO (Fig. 5d) were the common fertilization types, they were selected for further analysis according to soil type classification. It was shown that NP had the greatest effect on increasing maize yield in lithological soil and the lowest effect in cinnamon soil, NPK had the greatest effect on increasing maize yield in black soil and the lowest effect in chestnut soil, and the NPKO treatment had the greatest effect on increasing maize yield in fluvo-aquic soil and the lowest effect in brown loam soil.

### Response of WUE to fertilization

**Figure 6:**
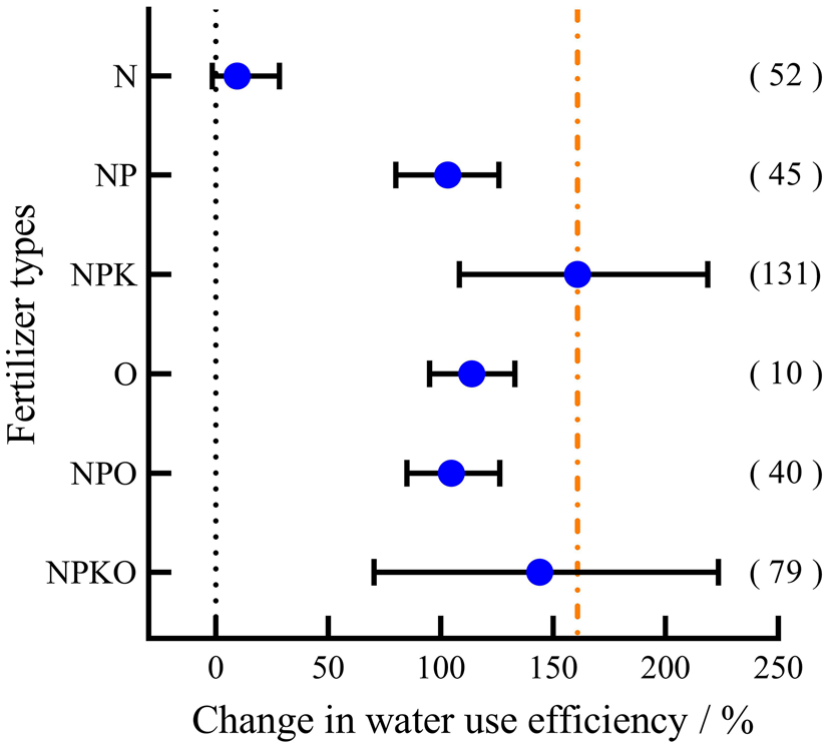
Response of WUE to different fertilization treatments.

Except N, which had no significant effect on WUE (P > 0.05, Fig. 6), other fertilization measures significantly improved the WUE of maize (P > 0.05) compared with no fertilization. The rate of change in water use efficiency from high to low was NPK (160.72%) > NPKO (144.05%) > O (113.83%) > NPO (104.72%) > NP (103.13%).

### Correlation analysis between the effects of fertilization measures on maize yield and climatic factors

**Figure 7:**
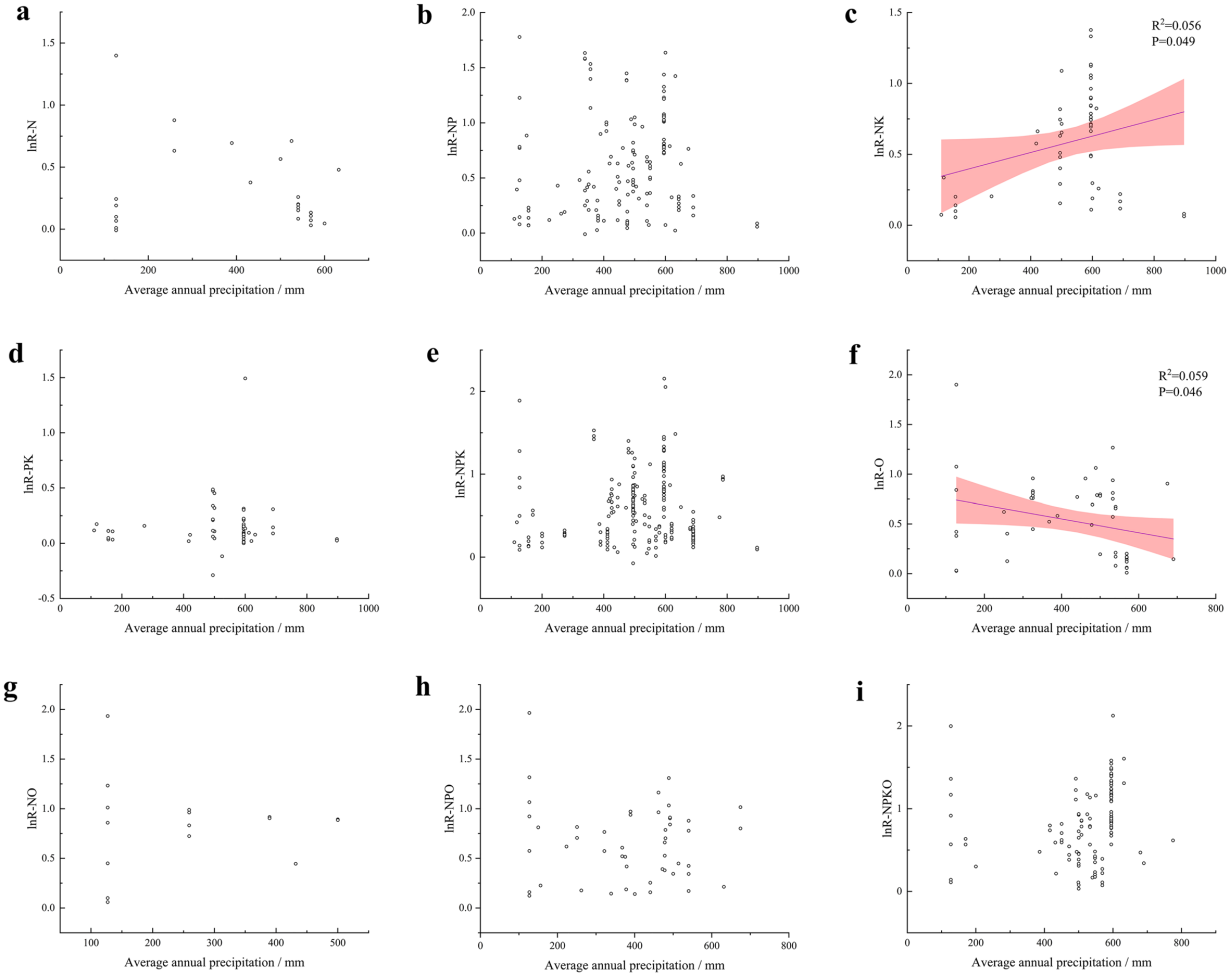
Linear regression analysis of effect sizes of maize yield and average annual precipitation under different fertilization practices. (**a**) N. (**b**) NP. (**c**) NK. (**d**) PK. (**e**) NPK. (**f**) O. (**g**) NO. (**h**) NPO. (**i**) NPKO.

**Figure 8:**
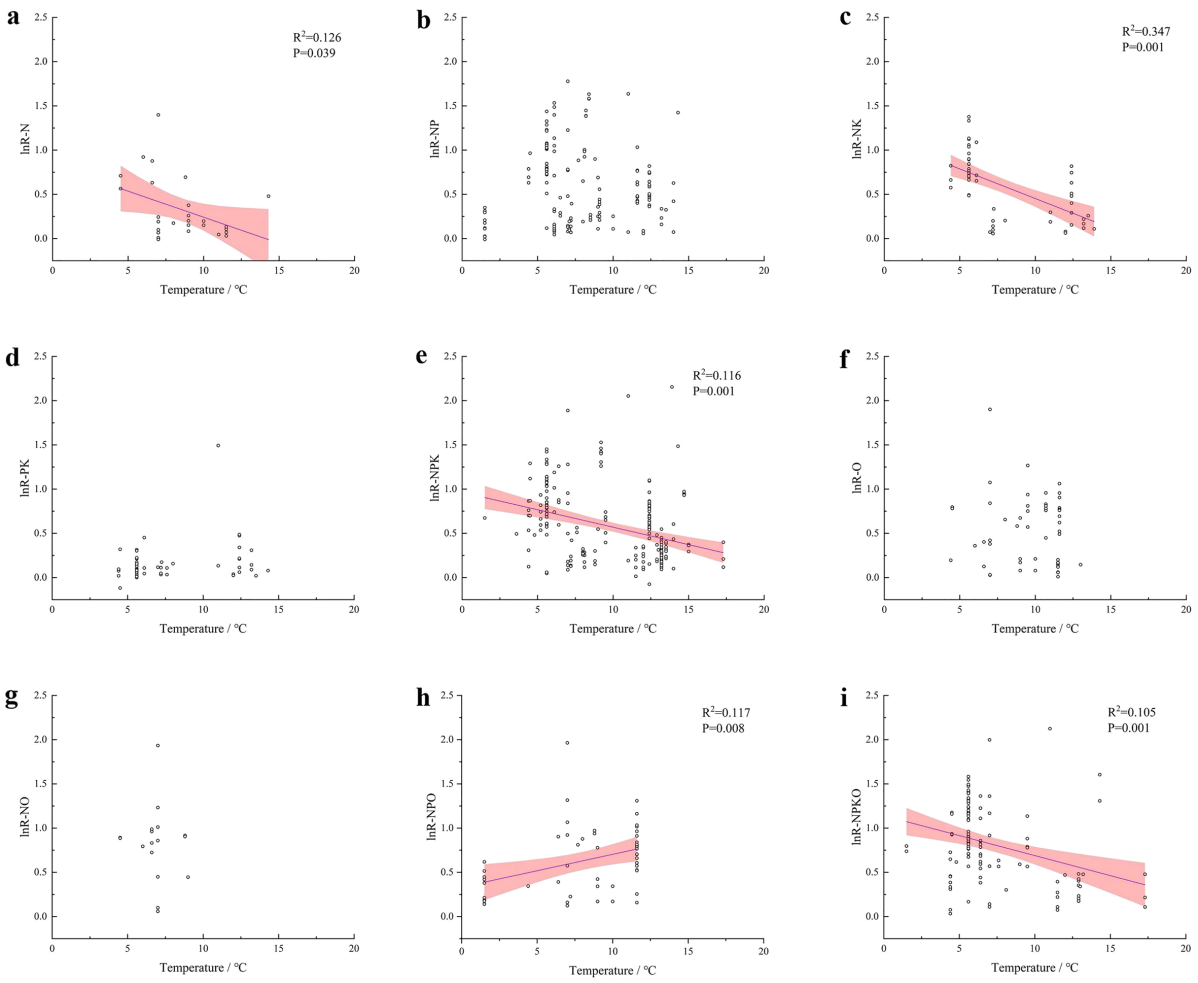
Linear regression analysis of effect sizes of maize yield and average annual temperature under different fertilization practices. (**a**) N. (**b**) NP. (**c**) NK. (**d**) PK. (**e**) NPK. (**f**) O. (**g**) NO. (**h**) NPO. (**i**) NPKO.

Linear regression was used to analyze the effects of average annual rainfall (Fig. 7) and average annual temperature (Fig. 8) on maize yield under different fertilization measures. The results showed that maize yield was positively correlated with annual average rainfall under the nitrogen and potassium fertilizer treatments (P < 0.05). Under the condition of organic fertilizer application alone, maize yield was significantly negatively correlated with average annual rainfall (P < 0.05). In terms of mean annual temperature, maize yield was significantly negatively correlated with mean annual temperature under the treatments of nitrogen fertilizer alone, NPK and organic fertilizer with NPK, and combined organic fertilizer (P < 0.05). There was a significant positive correlation between maize yield and mean annual temperature (P < 0.01) under the combination of nitrogen and potassium fertilizer. Under the condition of organic fertilizer combined with nitrogen fertilizer and phosphorus fertilizer, maize yield was positively correlated with mean annual temperature (P < 0.05).

## Discussion

### Effects of different fertilization measures on soil nutrients

Soil nutrients are an important basis for plant growth, and fertilization is one of the main exogenous input pathways to maintain their continuous balance. In this study, the integrated data analysis showed that under different fertilization measures, organic fertilizer combined with chemical fertilizer had the most obvious effect on improving soil nutrients. Although organic fertilizers do not provide more nutrients compared to chemical fertilizers, they need to be transformed and degraded before being taken up by plants compared to chemical fertilizers, which may be one of the driving factors for changes in soil microbial communities. At the same time, the neutral and alkaline nature of organic fertilizers prepared by the fermentation of manure or straw may limit the microbial activity in the original soil and the rate of decomposition of the organic matter, which is ultimately sequestered in the soil, while ensuring the supply of nutrients during the growing season and preventing nutrient loss^42,43^. In addition, the results of this study showed that the three fertilization measures, N, NP, and NPK without the application of organic fertilizers, increased the organic matter content of the soil from 2.36 to 14.62% compared to no fertilization (Fig. 4a). This may be due to the fact that chemical fertilizers are effective in providing specific nutrients to promote crop growth and increase crop yields, thereby increasing crop residue inputs and thus soil organic matter content, but they do not have the ability to increase soil organic matter and improve soil structure in the same way that organic fertilizers do^44,45^. Cai et al.^46^ found that the long-term application of organic fertilizer directly affected crop residues, increased soil organic carbon sequestration, and maintained the average annual growth rate of soil organic matter at 0.3–9.36 mg/hm^2^. Hou et al.^47^ found that by the third year, soil organic matter showed a downward trend when maize was grown without fertilization, and compared with the treatment of chemical fertilizer and no fertilization, the application of organic fertilizer could significantly increase soil organic matter content by 5.1–11.2% and 4.5–9.5%, respectively.

The soil nitrogen content is an important factor affecting plant rhizosphere growth and development. The higher total nitrogen content of soil under NPK fertilization or NPK combined with organic fertilizer application had a positive effect on maize root growth because enough nitrogen was added to the rhizosphere soil during maize growth, which increased the growth of roots and root exudates and then increased the recovered total nitrogen content and facilitated mineralization^48^. However, in the context of poor soil fertility, nitrogen application alone may increase the total nitrogen content, but improved maize growth may be significantly inhibited. This is because imbalanced fertilization reduces the nitrogen uptake by maize, which leads to the volatilization or leaching of nitrogen after application, while at the same time, not applying nitrogen fertilizer will lead to a significantly reduced total nitrogen content in the soil^49^.

When crops are harvested, phosphorus and nitrogen are removed from the soil. Therefore, fertilization without P would reduce the soil available P content. Pan et al.^50^ conducted a 22-year long-term experiment and showed that the application of nitrogen fertilizer alone reduced the content of soil available phosphorus, while the combined application of nitrogen and phosphorus fertilizer increased the content of soil available phosphorus by 2.49 and 2.77 times, compared with no fertilization and the application of nitrogen fertilizer alone, respectively. Meanwhile, some studies have found that NPK fertilization or NPK fertilization combined with organic fertilizer does not necessarily increase the soil available P content but instead reduces the soil P content in the year of application^51^. The reasons for this may be manifold, the neutral soil with a pH between 6–7 are ideal for crop growth, and the application of nitrogen, phosphorus and potash fertilizers can make the soil acidic because in strongly acidic or weakly alkaline soils, phosphorus forms complexes with aluminium, iron or calcium, which reduces the amount of phosphorus available for uptake by the crop, limiting the availability of phosphorus. And the soil itself is low in nutrients at this time and has not built up an effective phosphorus reservoir. Coupled with the crop's intensely rapid development and growth facilitated by fertilizers, the phosphorus it needs exceeds the amount of fertilizer supplementation, However, this phenomenon tends to increase significantly as long-term stable and balanced fertilization becomes total and effective phosphorus stable^52,53^. In addition, the effect of the depth of fertilizer application and microbial activity in the soil should not be ignored. On the one hand, an appropriate increase in the depth of phosphorus fertilizer application can regulate the growth and development of the root system, improve root distribution, and form more deep roots, thereby increasing the specific root surface area, which has a positive effect on delaying leaf senescence and enhancing photosynthetic performance^54^. On the other hand microorganisms can compete with plants for effective orthophosphate in the soil solution and also represent a large amount of fixed P that is temporarily unavailable to plants, and microbial phosphorus is a highly dynamic soil phosphorus pool and can change significantly in response to environmental factors such as soil temperature, humidity, and availability carbon, although ultimately microbial phosphorus has the potential to be utilized by plants^55^.

The long-term application of potassium fertilizer has a positive effect on the content of soil available potassium, but it can destroy the potassium and sodium balance in the soil and damage the soil structure^56^. In this study, there was no significant difference in the effects of the two fertilization measures on the soil available K content, and the soil available K content under the combined application of phosphorus and potassium was higher than that under the P-free treatment or the complete fertilizer treatment. The reason is that the potassium content absorbed during crop growth is higher than that applied by fertilizer^57^ or there is a change in adsorption sites in soil and the ability to fix potassium^58^.

In addition, the variation in soil nutrient content is not only affected by fertilization measures but is also determined by other factors. (1) The soil parent material refers to the original material from which the soil was formed and whose chemical and physical properties affect the content and availability of soil nutrients. Soils with different parent materials have different nutrient contents and ratios^59,60^. Huang et al.^61^ showed that the contents of total phosphorus and available phosphorus in the soil would increase or decreased with the application of P fertilizer, but the correlation analysis between the amount of P fertilizer applied and the available soil P content showed that the two were only weakly correlated (R^2^ = 0.25, P < 0.05, n = 108). Li et al.^62^ adopted the practice of not applying P and K fertilizers for 4 consecutive years to black soil with sufficient P and K reserves and found that this treatment did not affect the absorption of soil nutrients by maize. (2) The regional climate, mainly in terms of temperature and precipitation, both of which can change the root structure of crops by altering their photosynthesis and water uptake and thus impacting fertilizer use efficiency^63^. (3) Crop management, including fertilizer management (type, amount, timing and location of fertilizer application, etc.), cropping patterns (no-till, rotary, shallow and deep tillage, etc.) and yield enhancement measures (crop sets, rotations and intercropping, etc.), has an impact on plant‒soil-microbe relationships^64,65^.

### Effects of different fertilization measures on the soil water content

Fertilization, as one of the important means of artificially increasing yield, not only affects soil nutrients but also changes the soil water absorption capacity of plants during growth. Water scarcity negatively affects maize yield, which is contrary to the original intention of fertilization. Therefore, coupling water and fertilizer appropriately is the key to crop development in agroecosystems.

Our analysis showed that most fertilization treatments negatively affected the soil moisture under maize, especially N, NP, and NPO. First, these three fertilization treatments all involve the application of nitrogen fertilizer. Nitrogen can promote plant growth and development and is one of the essential nutrients for plant growth. Meanwhile, nitrogen is inextricably linked to plant root growth and transpiration because nitrogen is an important element in the synthesis of chlorophyll and proteins, and nitrogen fertilization promotes an increase in the leaf area of plants, which indirectly increases the transpiration area and may lead to an increase in the rate of transpiration, which is also a major factor driving the uptake of nutrients from the soil by the rhizosphere system during crop growth, leading to a decrease in soil moisture^66,67^. However, excessive nitrogen fertilization may lead to the disruption of soil granular structure, increase the risk of soil erosion, and may reduce soil permeability and water retention, which may be detrimental to the long-term productivity of the soil^68^. Second, the dosing of phosphorus fertilizers further meets the nutrient requirements for crop growth, leading to a rapid increase in plant leaf area, which in turn leads to a further increase in the rate of transpiration, resulting in more water being absorbed from the soil and evaporated into the atmosphere through the leaves of the plant^69^. Moreover, the application of phosphorus fertilizer makes plant hormones play a role in plant growth that should not be ignored, for example, under phosphorus stress, the gibberellin content in the root system increases, so that the original root structure changes thereby obtaining more effective phosphorus from the soil, while the soil is sufficient in phosphorus, gibberellin will be enriched in the leaves, which is conducive to the accumulation of dry matter^70^. Meanwhile, the auxin will rise with the application of phosphorus fertilizer with the growth of maize, and the higher the auxin level facilitates endosperm cell differentiation and dry matter transfer, and promotes the transport and distribution of photosynthetic products^71^.

Moreover, microorganisms in the soil are able to decompose organic matter to release water, while the application of nitrogen fertilizer may lead to a decrease in the biomass and activity of microorganisms, reducing the release of water^72,73^.

A single application of organic fertilizer steadily retained water in the 0–200 cm soil profile due to large amount of organic matter in the organic fertilizer, which strengthened the soil aggregate stability^74,75^. The emergence of this phenomenon not only shows that the soil organic matter content has a positive effect on the soil water-holding capacity but may also be related to the soil organic matter content before fertilization^76,77^. Factors affecting differences in the initial soil organic matter content are multifaceted and are related to soil pH and soil management practices, in addition to the influence of soil type^78,79^. On the one hand, soil pH has an effect on the decomposition rate and stability of organic matter. In acidic soil, the decomposition rate of organic matter is slower, and the organic matter content is relatively high. In alkaline soil, the decomposition rate of organic matter is faster, and the organic matter content is relatively low^80,81^. On the other hand, the application of organic fertilizers, tillage and the choice of tillage methods can affect the accumulation and decomposition of soil organic matter. In addition, the results showed a decreasing trend in the soil water content when organic fertilizers were combined with nitrogen, phosphorus and potassium fertilizers, which might be because fertilizers increase the salt content in the soil, thereby loosening the soil particles, thus destroying the soil structure and decreasing the permeability of the soil, which led to a decrease in the soil water content. However, we cannot exclude the possibility that the plant root system will increase the absorption of water and nutrients due to fertilizer application, which promotes its own growth and at the same time increases the evaporation of water, thus leading to a decrease in the soil water content^82^.

### Effects of different fertilization practices on maize yield and WUE

This study showed that the percentage increase in yield from a single organic fertilizer application was slightly higher than or close to that of a single chemical fertilizer application, but both were lower than that of the combined organic and chemical fertilizer application. This is because organic fertilizer can not only reduce soil bulk density but also increase soil porosity, which significantly improves plant nutrient absorption^83^. In addition, the combined application of organic fertilizer and inorganic fertilizer can effectively coordinate the rapid release of inorganic nutrients and the slow supply of organic nutrients in the soil to meet the nutrient demand for crop growth and thus improve crop yield^84,85^.Moreover, according to the results in Fig. 8, maize yield is more sensitive to temperature, and shows a decreasing trend with increasing temperature. This may be due to the fact that summer maize is mainly grown in China, and the average temperature during its reproductive period is relatively high. A certain degree of decrease in the maximum daytime temperature not only does not reduce the intensity of photosynthesis, but also reduces the risk of high temperature and drought, which helps to increase maize yield^86,87^. Among them, the increase in maize yield under NPO measures showed an increasing trend with increasing temperature, which could be attributed to the small increase in temperature promoting microbial activity in the soil, and the rich organic matter in organic fertilizers providing a food source for soil microbes, which helps the microbes to decompose the organic matter and release more nutrients to the plants^88^. Meanwhile, under different fertilization treatments, according to the effect of soil type on maize yield, the combination of N and P fertilization should be the primary fertilizer treatment to ensure the continuous increase in maize yield for soil types in Northwest China, which are mostly lithologic soil. Most of the soils in Northeast and North China are black soil and fluvo-aquic soil, respectively, which should be treated with an all-nutrient combined application or organic fertilizer combined with all-nutrient chemical fertilizer. According to the linear regression analysis of maize yield and climate factors, Northeast China and part of North China with lower temperatures and higher rainfall are more suitable for maize cultivation.

In addition, rational fertilization measures are key to improving crop WUE.And there is a close physiological relationship between nutrient uptake and transpiration. First, nutrient uptake is the process by which plants absorb nutrients from the soil. Plants absorb water and nutrients, including essential elements such as nitrogen, phosphorus and potassium, through the root system. These nutrients are essential for plant growth and development, especially nitrogen, which is involved in many biochemical processes in plants and also plays an important role in increasing corn kernel yields^89^. Second, phosphorus promotes root development, expands the surface area of the root system for soil nutrient uptake, and improves corn's ability to absorb soil nutrients and water, which is an important factor in increasing corn's below-ground biomass^90,91^. Additionally, potassium is involved in many physiological processes within the maize plant including osmotic regulation, enzyme activation, and photosynthesis product transport, and improves maize tolerance to biotic and abiotic stresses^92^. Adequate potassium supply can help the crop maintain a high photosynthetic capacity while reducing unnecessary water loss, therefore increasing WUE and contributing to overall crop health and yield^93^. Finally, the application of organic fertilizers can improve soil structure and texture, increase soil water retention and fertility, and improve soil nutrient availability. Organic fertilizers are rich in organic matter and microorganisms, which can improve the physical, chemical and biological properties of the soil, promote soil microbial activities and nutrient transformation, and increase the effectiveness and availability of nutrients, thus improving the efficiency of nutrient and water use by the crop^94^. In this study, the improvement of corn yield and WUE under NPKO treatment had a good performance, which may be due to the improvement of soil characteristics through the reasonable fertilizer application measures, optimizing soil nutrient supply to meet the crop's nutrient needs. soil characteristics and optimize soil nutrient supply to meet the crop's nutrient needs, thus increasing yield and WUE. Moreover, reasonable fertilization measures can also adjust the pH of the soil to provide suitable environmental conditions conducive to the absorption of nutrients by the plant root system. Different plants have different adaptabilities to soil pH, and a reasonable adjustment of the soil pH can provide a suitable environment to improve the effectiveness and availability of nutrients and increase the nutrient absorption capacity of plants^95^.

In this study, we investigated the effects of different fertilization measures on maize yield, soil nutrients, soil moisture and water use efficiency in northern China through meta-analysis and elaborated on the intrinsic mechanisms that may affect the changes in plant physiology and the soil environment before and after fertilization. This study provides a basis for subsequent studies to compare and summarize the effects and potential mechanisms of different fertilization measures. This helps to gain a deeper understanding of the effects of fertilizer application on crop yield and soil quality and provides guidance for further research. Meanwhile, management can also formulate appropriate agricultural policies based on the results of this study to help farmers choose appropriate fertilizer application measures to improve the efficiency of agricultural production and the sustainability of the farming system.

## Conclusion

Based on the data obtained in this meta-analysis, from the long-term perspective of maintaining farmland ecosystems, ensuring the supply of soil nutrients to crops and balancing soil water, fertilization practices combining organic fertilizer with chemical fertilizer should be adopted in northern China, combined with water conservation measures such as straw return and film mulching, and appropriate tillage measures should be applied according to different geographical locations to achieve the sustainable management of farmland soil in China (Supplementary information).

### Supplementary Information

Supplementary Information.

## Data Availability

Available upon request to the corresponding author.
