# Supplementary material for: Effects of different fertilization practices on maize yield, soil nutrients, soil moisture, and water use efficiency in northern China based on a meta-analysis
Source: Sci Rep. 2024 Mar 18;14:6480. doi: 10.1038/s41598-024-57031-z (PMC10948899; doi:10.1038/s41598-024-57031-z)
Supplement: Supplementary file 1 — Supplementary Information. [file 41598_2024_57031_MOESM1_ESM.pdf]

# Effects of different fertilization practices on maize yield, soil nutrients, soil moisture, and water use efficiency in northern China based on a meta-analysis

Minghao Jiang [1], Chao Dong [2], Wenpeng Bian [1], Wenbei Zhang [1], and Yong Wang [1,3,*]

1 School of Water Conservancy and Civil Engineering, Northeast Agricultural University, Harbin, 150030, China

2 Inner Mongolia University of Technology, Huhhot, Inner Mongolia 010051, China

3 College of Horticulture and Landscape Architecture, Northeast Agricultural University, Harbin 150030, China

*Correspondence: wangyong@neau.edu.cn

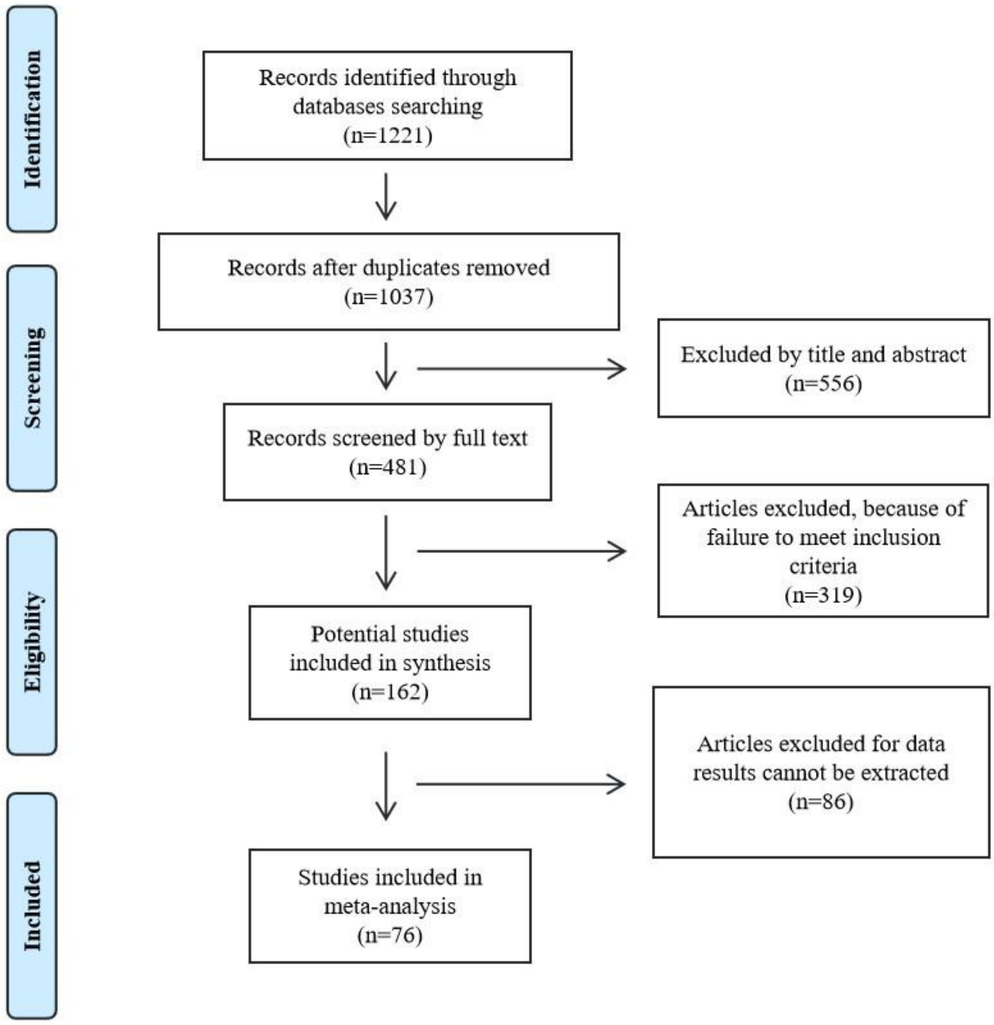


**Figure S1.** Flow diagram of the literature search and screening.

**Table S1.** The detailed information of selected studies and collected data. The data for authors, year, type of fertilization and indicator.

| Authors | Type of fertilization | Indicator (Sample Size) | | | | | | | Ref. |
|---|---|---|---|---|---|---|---|---|---|
| | | Soil moisture | Soil nutrients | | | | Yield | Water use efficiency | |
| | | | Organic matter | Total nitrogen | Available phosphorus | Available potassium | | | |
| Li (Li,Long) et al., 2022 | NP, NK, PK, NPK | | 12 | | 12 | 12 | 5 | 5 | [1] |
| Yu et al., 2021 | NPK, O, NPKO | | 25 | 25 | 25 | 25 | 25 | | [2] |
| Wu (Wu, Kesheng) et al., 2021 | NP, PO, NPO | | 4 | 4 | 4 | 4 | 4 | | [3] |
| Jiang et al., 2021 | NPK, NPKO | | | | 6 | 6 | 6 | | [4] |
| Chou et al., 2019 | NP, NK, PK, NPK | | | 15 | 15 | 15 | 15 | | [5] |
| Li et al., 2019 | NPK, NPKO | | 9 | 9 | 9 | 9 | 3 | | [6] |
| Huang et al., 2018 | N, NP, O, NO, NPO | | 8 | 8 | 8 | 8 | 8 | | [7] |
| Wang et al., 2018 | NPK | | 2 | 2 | 2 | 2 | 2 | | [8] |
| Zhang et al., 2017 | NPK | | | 18 | 18 | 18 | 18 | | [9] |
| Li et al., 2017 | NP, NK, PK, NPK | | | | | | 20 | | [10] |
| Yang et al., 2016 | NPK | | | | 8 | 8 | 4 | | [11] |
| Jiang et al., 2016 | NPK, PKO, NPKO | | | | 5 | 5 | 5 | | [12] |
| Xing et al., 2016 | NPK, NPKO | | 16 | 16 | 16 | 16 | 16 | | [13] |
| Hua et al., 2016 | NPK | | | | 4 | 4 | 4 | | [14] |
| Gao et al., 2015 | NP, NK, PK, NPK, NPKO | | | | | | 161 | | [15] |
| Li (Li, Hui) et al., 2015 | N, PK, NPK, O, NO, PKO, NPKO | | | | | | 12 | | [16] |
| Li (Li, Boyu) et al., 2015 | NP | | | | | | 8 | | [17] |
| Ma et al., 2015 | NP, NK, PK, NPK | | | | | | 40 | | [18] |
| Zhu et al., 2015 | NP, NPO | | 3 | 3 | 3 | 3 | 3 | | [19] |
| Hao et al., 2015 | NPK, O, NPO, NPKO | | | | | | 5 | | [20] |
| Huo et al., 2015 | N, O, NO | | 9 | 9 | 9 | 9 | 9 | | [21] |
| Wang (Wang, Xiukang) et al., 2015 | NP | | | | | | 9 | 9 | [22] |
| Pan et al., 2014 | NP, NK, PK, NPK | | | | | | 14 | | [23] |
| Zhang et al., 2013 | N, P, NP, O, NPO | | | | | | 24 | | [24] |
| Ma et al., 2012 | NP, NK, PK, NPK | | | | | | 15 | | [25] |
| Jiao et al., 2012 | NPK | | | | | | 16 | | [26] |
| Liu et al., 2011 | NP, NPO | | 4 | 4 | 4 | 4 | | | [27] |
| E et al., 2010 | N, NP, NPK, O, NO, NPO, NPKO | | | | | | 56 | | [28] |

| | | | | | | | | | |
|---|---|---|---|---|---|---|---|---|---|
| Sun et al., 2010 | N, O, NO, NPO | | 6 | 6 | 6 | 6 | | | [29] |
| Liu et al., 2009 | NP, NK, PK, NPK | | | | | | 14 | | [30] |
| Feng et al., 2009 | NP, NK, PK, NPK | | | | | | 5 | | [31] |
| Li et al., 2009 | NP, NK, PK, NPK | | | | | | 64 | | [32] |
| Liu et al., 2007 | N, NP, NK, PK, NPK, NPKO | | 7 | 7 | 7 | 7 | 7 | | [33] |
| Hou et al. 2022 | NP, NPK | | | | | | 9 | 9 | [34] |
| Wang (Wang, Yilun) et al., 2015 | NPK | | | 8 | 8 | 8 | 8 | | [35] |
| Jiao et al., 2022 | NPK, NPKO | | | | | | 6 | | [36] |
| Xie et al., 2022 | NPK, NPKO | | 6 | 6 | | | 12 | | [37] |
| Tao et al., 2022 | NPK, NPKO | | | | | | 4 | | [38] |
| Li (Li, Yali) et al., 2022 | PK, NPK, NPKO | | | | | | 8 | | [39] |
| Liu et al., 2022 | NPK, NPKO | | | | | | 3 | | [40] |
| Song et al., 2022 | NPK | | | 2 | 2 | 2 | 2 | | [41] |
| Shi et al., 2021 | NP, NK, PK, NPK | | | | | | 10 | | [42] |
| Liu et al., 2021 | NP | | 4 | 4 | | | 8 | | [43] |
| Wu (Wu, Zhiyuan) et al., 2021 | O | 7 | | | | | 7 | | [44] |
| Lv et al., 2020 | NPK | | | | | | 5 | | [45] |
| Cao et al., 2020 | NPK, O, NPKO | | | | | | 20 | | [46] |
| Guo et al., 2020 | NP, NK, PK, NPK | | | | | | 5 | | [47] |
| Wang et al., 2019 | NPK ,NPKO | | | | | | 3 | | [48] |
| E et al., 2019 | N, NP, O, NPO | | 5 | 5 | 5 | 5 | 5 | | [49] |
| Xin et al., 2021 | NK, NPK, PKO | | | | | | 5 | | [50] |
| Xu et al., 2021 | NPK, O, NPKO | | | | | | 4 | | [51] |
| Liang et al., 2021 | NPK, NPKO | | 3 | 3 | 3 | 3 | 3 | | [52] |
| Zhang (Zhang, Yunlong) et al., 2016 | NPK, NPKO | | 9 | | 9 | 9 | 9 | | [53] |
| Mia et al., 2015 | N, NP, PK, NPK, NPKO | 3 | | | | | 14 | | [54] |
| Zou et al., 2012 | NP, NPO | | | | | | 30 | | [55] |
| Chen et al., 2021 | NPK, NPO, NPKO | 108 | | | | | 12 | 12 | [56] |
| Zhang et al., 2012 | NP | 4 | | | | | 4 | 4 | [57] |
| Zhang et al., 2015 | NPK | 15 | | | | | | | [58] |
| Gao et al., 2015 | NPK, NPKO | 3 | | | | | 3 | 3 | [59] |
| Pang et al., 2021 | NP | 220 | | | | | 20 | 20 | [60] |
| Zheng et al., 2019 | NPK | 120 | | | | | 8 | 8 | [61] |

| | | | | | | | | |
|---|---|---|---|---|---|---|---|---|
| Guo et al., 2015 | NP, NPK | 90 | | | | | 9 | 9 | [62] |
| Zhang (Zhang, Wanqing) et al., 2016 | N | | | | | | 6 | 6 | [63] |
| Wang et al., 2016 | NP | 30 | | | | | | | [64] |
| Wang et al., 2017 | N, P, NP, O, NPO | | | | | | 12 | | [65] |
| Wang (Wang, Xiaojiao) et al., 2021 | N, O, NO | | | | | | 4 | | [66] |
| Zou et al., 2012 | NP, NPO | | | | | | | 12 | [67] |
| Zhou et al., 2004 | NP, O, NPO | | | | | | 50 | 50 | [68] |
| He et al., 2018 | NPK, NPKO | | 5 | 5 | 5 | 5 | | | [69] |
| Chen et al., 2012 | N, NPK, NO, NPKO | 50 | | | | | 5 | 5 | [70] |
| Zhang et al., 2021 | NP | 200 | | | | | 10 | 20 | [71] |
| Wang (Wang, Xiquan) et al., 2021 | NPK, NPKO | 40 | | | | | | | [72] |
| Peng et al., 2020 | N | 40 | | | | | | | [73] |
| Hu et al., 2017 | N | 70 | | | | | | | [74] |
| Shu et al., 2022 | N, NO | 36 | | | | | | | [75] |
| Zhang, 2022 | N | 25 | | | | | | | [76] |

**Table S2.** The detailed information of selected studies and collected data. The data for pH and soil physiochemical properties before the application of the treatments.

| References | pH | Organic matter (g/ kg) | Total nitrogen (g/ kg) | Available phosphorus (mg/ kg) | Available potassium (mg/ kg) | Bulk density (g/ $cm^3$) |
|---|---|---|---|---|---|---|
| 1 | 8.33 | 16.3 | | 4.45 | 118.59 | 1.38 |
| 2 | 8.3~8.5 | 9.87 | 0.640 | 31.75 | 271.25 | |
| 3 | 8.8 | 16.35 | 1.06 | 13.1 | 180 | 1.40 |
| 4 | 6.9 | 33.03 | 1.70 | 26.4 | 142.75 | |
| 5 | 6.9 | 33.4 | 1.8 | 40.2 | 252.7 | |
| 6 | 8.2 | 7.14 | | 18.08 | 130.46 | 1.12 |
| 7 | 8.2 | 5.64 | 0.45 | 2.69 | | |
| 8 | 8.20 | 25.96 | 1.72 | 19.3 | 143.81 | |
| 9 | 6.54 | 21.64 | 1.28 | 15.61 | 84.23 | |
| | 6.92 | 18.35 | 1.15 | 14.28 | 97.62 | |
| 10 | 7.8 | 16.9 | 0.71 | 26 | 192.5 | |
| | 7.8 | 16.4 | 0.75 | 26.4 | 195.8 | |
| | 8.3 | 15.1 | 0.98 | 18.5 | 200.7 | |
| | 8.0 | 12.2 | 0.65 | 24.8 | 214.0 | |
| 11 | 8.13 | 15.20 | 1.68 | 12.64 | 117.0 | |
| 12 | 5.37 | 17.6 | | 36.18 | 175.59 | |
| 13 | 8.3 | 20.3 | 1.2 | 37.4 | 282 | |
| 14 | | 13.45 | | 46.45 | 198.72 | |
| 15 | 7.6 | 22.8 | 1.4 | 27.0 | 190.0 | |
| 16 | | 27.79 | 1.90 | 27.0 | 190 | |
| 17 | | 25.96 | 1.19 | 37.59 | 161.17 | |
| 18 | | 12.4 | | 30.1 | 189.9 | |
| 19 | 8.4 | 23.2 | 1.214 | 45.7 | 275 | |
| 20 | 7.2 | 15.5 | 1.47 | 51 | 200 | |
| 21 | 8.25~8.74 | 8.01 | 0.59 | 6.5 | 193 | 1.43 |
| 22 | 8.4 | 10.5 | 0.8 | 4.58 | | 1.28 |
| 23 | 8.45 | 14.96 | 1.06 | 4.8 | 199.0 | |
| 24 | | 11.75 | | 13.94 | 150.43 | 1.22 |

| | | | | | | |
|---|---|---|---|---|---|---|
| 25 | 8.14 | 14.3 | 0.9 | 26.8 | 163 | |
| 26 | | 13.95 | 0.89 | 17.23 | 95.50 | 1.45 |
| 27 | 7.7 | 13.5 | 0.79 | 16.23 | 146.05 | |
| 28 | | 20.80 | 0.76 | 21.70 | 99.10 | |
| 29 | | 13.5 | 0.791 | 16.23 | 146.05 | |
| 30 | | 7.7 | | 10.7 | 61.5 | |
| 31 | | 12.907 | 0.8424 | 30.1 | 86 | |
| 32 | 8.48 | 6.1 | 0.34 | 9.29 | 52 | |
| 33 | 8.22 | 12.31 | 0.81 | 4.62 | 65.27 | |
| 34 | 7.3 | 15.80 | 0.95 | 10.39 | 166 | 1.53 |
| 35 | | 10.4 | | 19.5 | 99.0 | |
| | | 12.0 | | 29.2 | 191.0 | |
| 36 | 6.3 | 29.36 | 1.52 | 42.64 | 166.83 | |
| 37 | 8.36 | 11.9 | 0.8 | | | |
| 38 | 7.60 | 16.41 | 0.95 | 11.43 | 411 | |
| 39 | 8.1 | 14.9 | | 10.2 | 146 | |
| 40 | 7.24 | | 0.9 | 12.01 | | |
| 41 | | 21.6 | 1.23 | 21.9 | 161.2 | |
| 42 | 7.27 | 116.67 | | 19.59 | 93.21 | |
| | 7.36 | 138.28 | | 23.78 | 129.12 | |
| 43 | 8.7 | 11.5 | 0.83 | 12.3 | 109.8 | |
| 44 | 8.27 | 9.2 | 0.62 | 6.41 | 113.22 | |
| 45 | 6.8 | 15.98 | | 22.80 | 136.9 | |
| 46 | 8.3 | 7.41 | 0.50 | 2.01 | 106.85 | |
| 47 | 8.40 | 43.9 | | 42.65 | 137.35 | |
| 48 | 6.1 | 23.03 | 2.52 | 23.36 | 75.15 | |
| 49 | 8.20 | 10.75 | 0.95 | 6.77 | 163.2 | |
| 50 | 8.66 | 5.83 | 0.44 | 1.92 | 78.7 | |
| 51 | 7.9 | 15.2 | 1.40 | | 120 | |
| 52 | 6.2 | 27.07 | 180 | 17.9 | 149 | 1.42 |
| 53 | 7.24 | 9.0 | 13.7 | 12.01 | 176.22 | |
| 54 | 8.30 | 10.2 | 0.65 | 7.7 | 65 | |
| | 7.6 | 23.3 | 1.4 | 27.0 | 190 | |

| | | | | | | |
|---|---|---|---|---|---|---|
| 55 | | 27.8 | | | | |
| 56 | 8.36 | 11.92 | 0.78 | | | 1.17 |
| 57 | | 10.5 | 1.12 | 6.41 | 125.0 | |
| 58 | 5.2 | 26.4 | 1.23 | 15.2 | 101 | |
| 59 | 7.6 | 23.3 | 1.40 | 27 | 190 | |
| 60 | 8.4 | 10.73 | 1.19 | 12.6 | 161.2 | 1.33 |
| 61 | 8.4 | 6.50 | 0.80 | 5.0 | 129.3 | 1.35 |
| 62 | | 12.58 | 0.86 | 13.32 | 138 | 1.15 |
| 63 | | | | | | 1.57 |
| 64 | 8.0 | 16.2 | 0.97 | 0.66 | 130.4 | 1.13 |
| 65 | | 10.71 | | 14.28 | 110.75 | 1.33 |
| 66 | 8.40 | 7.46 | 0.81 | 11.90 | 330.20 | 1.29 |
| 67 | | | | | | |
| 68 | 8.4 | 23.5 | 1.07 | 4.97 | 117.2 | |
| 69 | 7.6 | 22.8 | 1.4 | 11.79 | 158.33 | 1.19 |
| 70 | 8.14 | 8.22 | | 11.4 | 137.2 | 2.65 |
| 71 | | 12.75 | 0.74 | 4.3 | 100.0 | |
| 72 | 7.74 | 15.5 | 1.3 | 89.8 | | 1.31 |
| 73 | 8.3 | 12.86 | 0.85 | 11.5 | 330.2 | 1.29 |
| 74 | | 2.12 | 0.88 | 13.67 | 182.30 | 1.38 |
| 75 | 8.10 | 4.31 | 0.26 | 3.31 | 66.49 | 1.40 |
| 76 | | | | 46.08 | 140.21 | 1.40 |

**References**


1. Li, L.; Xiao, R.; Zhang, Y. Effects of Combined Application of Nitrogen, Phosphorus and Potassium on Seed Maize Yield and Economic Benefit. *Crops* **2022**, *210*, 111-117.
2. Yu, Y.; Guo, N.; Yan, S.; Jiang, Y.; Han, B.; Wu, W.; Jia, X. Effects of substitution of chemical fertilizers by organic fertilizer on soil fertility and maize yield. *Soil Fert. Sci. China* **2021**, *3*, 148-154.
3. Wu, K.; Che, Z.; Bao, X.; Zhang, J.; Lu, B.; Yang, X.; Yang, R. Analysis on Soil Fertility Characteristics and Crop Yield Sustainability of Irrigated Desert Soil with Long-term Organic Fertilization Application. *J. Soil Water Conserv. China* **2021**, *35*, 333-340.
4. Jiang, B.; Wang, Y.; Zhang, J. Effect of nitrogen reduction combined with humic acid application on physical and chemical properties of black soil and maize yield *J. Northeast Agric. Univ. (Chin. Ed.)* **2021**, *52*, 29-36.
5. Chou, S.; Li, N.; He, P.; Wei, D.; Jin, L.; Zhao, S.; Xu, X.; Zhou, W. Nutrients Use Efficiency Change of Chemical Fertilizers for Spring Maize in a Typical Black Soil *Sci. Agric. Sin.* **2019**, *52*, 2824-2834.
6. Li, Q.; Wang, G.; Xie, Z.; Liu, Z.; Liang, F. Effects of Additional Microbial Fertilizer on Soil Microbe Amount and Soil Enzyme Activity in Maize under Mulched Drip Irrigation. *J. Maize Sci.* **2019**, *27*, 141-147.
7. Huang, X.; Wang, L.; Huang, X.; Zhao, C.; Liu H. Sustainability of soil-crop systems under different long-term fertilizations in Chestnut Cinnamon soil areas. *Chin. J. Eco-Agric*. **2018**, *26*, 1107-1116.
8. Wang, Z.; Tang, C.; Zhao, C.; Yang, K.; Li, Z.; Wang, H.; Yin, D. Effects of Biochar and Chemical Fertilizer Interaction on Soil Nutrient and Maize Yield *J. Maize Sci.* **2018**, *26*, 146-151+159.
9. Zhang, L.; Wang, L.; Kong, L.; Yang, J.; Xie J.; Hou, Y. Nutrient Utilization and Soil Nutrient Balance of Spring Maize under Different Fertilizer Application Modes. *Chin. J. Soil Sci*. **2017**, *48*, 1169-1176.
10. Li, Y.; Wang, X.; Feng, Y.; Hu, F.; Lu, J.; Bai, B.; Liu, Q. Research on the Abundance and Deficiency Indices of Soil Nutrients for Maize in a Water Sand Land in the Middle Area of Gansu. *Chin. J. Soil Sci*. **2017**, *48*, 182-189.
11. Yang, F.; Yan, Q.; Lu, J., Li, F.; Wang, M.; Dong, F. Effects of Different Fertilizer Treatments under Equal Nitrogen Level on the Yield, Soil Nutrient Uptake and Utilization of Summer Maize. *J. Maize Sci.* **2016**, *24*, 136-143.
12. Jiang, B.; Hou, L.; Gao, Q.; Deng, H.; Xie, X.; Guo, T. Effect of organic-inorganic fertilizer on soil microbial, enzymes and maize yield. *J. Northeast Agric. Univ. (Chin. Ed.)* **2016**, *47*, 37-45.
13. Xing, P.; Gao, S.; Ma, M.; Zhou, X.; Zhao, T.; Sun, J.; Shen, D. Impact of organic manure supplement chemical fertilizer partially on soil nutrition,enzyme activity and crop yield in the north China plain. *Soil Fert. Sci. China* **2016**, *263*, 98-104.
14. Hua, R.; Li, J.; Wang, L.; Li, D.; Feng, L.; Dong, Y. Effects of Different Fertigation Strategies on Maize Yield,Nutrient Uptake,and Economic Benefit. *Xinjiang Agric. Sci.* **2016**, *53*, 68-76.
15. Gao, H.; Peng, C.; Zhang, X.; Li, Q.; Zhu, P. Effect of Long-Term Different Fertilization on Maize Yield Stability in the Northeast Black Soil Region. *Sci. Agric. Sin.* **2015**, *48*, 4790-4799.
16. Li, H.; Xu, M.;Zhu, P.; Zhang, W.; Zhang, H.; Li, Z. Change of nitrogen use efficiency of maize affected by long-term manure fertilization in the typical black soil. *J. Plant Nutr. Fert*. **2015**, *21*, 1506-1513.
17. Li,B.; Li, Y.; Zhang, P.; Jia, Z.; Wang, J.; Ren, X. Effect of fertilization under root-zone rainfall micro-collecting planting pattern of maize in semi-arid region. *Agric. Res.Arid Areas* **2015**,

*33*, 52-57.

18. Ma, Z.; Zhang, X.; Zhou, C.; Ma, L.; Wang, L.; Wang, Y.; Ma, Z. Abundant/lack index of nitrogen,phosphorus,and potassium for maize in Western Guanzhong and determination of economic optimum fertilization rate. *J. Northwest Sci-Tech Univ. Agric. For.(Nat.Sci. Ed.)* **2015**, *43*,145-151.
19. Zhu, Y.; Wang, U.; Peng, Y.; Gong, X.; Jin, X.; Zhou, J. Changes of Soil Nutrients and Microbial Communities under the Condition of Organic Fertilizers Replacing Part of Chemical Fertilizers. *Chin. J. Soil Sci*. **2015**, *46*, 1161-1167.
20. Hao, X.; Zhou, B.; Ma, X.; Gao, Z. Characteristics of crop yield and nutrient balance under different long-term fertilization practices in black soil. *Trans. Chin. Soc. Agric. Eng.* **2015**, *31*, 178-185.
21. Huo, L.; Wang, C.; Pang, H.; Yang, S.; Li, Y.; Jiang, W. Effects of combined application of organic and inorganic fertilizers on physical and chemical properties and crop yields in alkali-saline soil. *Agric. Res. Arid Areas* **2015**, *33*, 105-111.
22. Wang, X.; Li, Z.; Xing, Y. Effects of mulching and fertilization on maize yield,soil temperature and nitrate- N distribution. *J. Plant Nutr. Fert.* **2015**, *21*, 884-897.
23. Pan, J.; Xiao, H.; Wang, Y.; Cheng, W.; Yu, C.; Lu, W. Study on Fertilization Effects on Maize and Soil Nutrient Supply Capacity in Coastal Saline Areas. *Acta Agric. Boreali-Sin.* **2014**, *29*, 208-213.
24. Zhang, J.; Wang, Y.; Fan, T.; Guo, T.; Zhao, G.; Dang, Y.; Wang, L.; Li, S. Effects of different tillage and fertilization modes on the soil physical and chemical properties and crop yield under winter wheat/spring corn rotation on dry land of east Gansu, Northwest China. *Chin. J. Appl. Ecol.* **2013**, *24*, 1001-1008.
25. Ma, J.; Tian, S.; Tian, H.; Mao, H.; Wang, J.; Dong, P. Abundance and Deficiency Status of Silt Soil Nutrient Study in Bank of North Yellow River Irrigation District of Ningxia. *Chin. Agric. Sci. Bull.* **2012**, *28*, 236-242.
26. Jiao, Y.; Meng, X.; Ma, X.; Feng, R. Effects of Nitrogen Application on the Yields of Wheat and Maize and the Distribution of Soil Nutrients. *South-to-North Water Transfers Water Sci. Technol*. **2012**, *10*, 103-108.
27. Liu, M.; Sun, J.; Li, L.; Liu, J.; Zhang, X. Effect of Fertilizer Treatments on Soil Microbe Amount and Nutrition Content in Rhizosphere of Silage Maize. *Chin. J. Soil Sci*. **2011**, *42*, 816-821.
28. E, S.; Yang, S.; Guo, Y.; Suo, D.; Yang, S.; Cui, Y.; Wang, B. Effects of long-term fertilization on crop yield and indigenous soil nutrient supply in Hexi Oasis of Gansu Province. *J. Plant Nutr. Fert*. **2010**, *16*, 786-793.
29. Sun, J.; Liu, M.; Li, L.; Liu, J. The Effect of Different Fertilization Treatments on Soil Physical and Chemical Property. *Acta Agric. Boreali-Sin*. **2010**, *25*, 221-225.
30. Liu, Z.; Feng, Y.; Hu, D.; Su, E.; Zhao, R.; Zhang, L.; Sun, C. Effects of soil nutrient supply level and different fertilizer application rates on maize yield. *Journal of Northern Agriculture* **2009**, *2*, 31-34.
31. Feng, Z.; Wang, S.; Zhang, S.; Zhang, Z. Investigation of Restrict Yield Nutrient of Spring Corn in Tangshan in Hebei Province. *Chin. Agric. Sci. Bull*. **2009**, *25*, 291-294.
32. Li, X.; Men, M.; Wang, S.; Qi, Y.; Xu, H. The effects of long-term fertilization on crop yields and farmland nutrient equilibrium. *Acta Pratac. Sin.* **2009**, *18*, 9-16.
33. Liu, E.; Zhao, B.; Hu, C.; Li, X.; Li, Y. Effects of long-term nitrogen,phosphorus and potassium fertilizer applications on maize yield and soil fertility. *J. Plant Nutr. Fert*. **2007**, *59*, 789-794.
34. Hou, Y.; Huang, M.; Fu, G.; Li, Y.; Tian, W.; Li, J.; Wu, J.; Zhao, K.; Zhang, Z.; Zhao, Z.; Lv, J.; Yao, Y. Effects of Straw Return Replacing the Application of Potassium Fertilizer on Crop Productivity and Soil Nitrate Accumulation in Dry land Summer Maize-Winter Wheat Rotation System. *J. Soil Water Conserv. China* **2022**, *36*, 311-318.
35. Wang, Y.; Lu, Y.; Liu, J.; Su, R.; Ma, L.; Tan, J.; Bai, Y. Effects of special slow-release fertilizer on yield and nutrient absorption and utilization of summer maize. *Soil Fert. Sci. China* **2015**, *255*, 29-32.

36. Jiang, B.; Liu, L.; Liu, J.; Yu, S.; Liang, Y.; Shen, H.; Shao, H. Effects of dairy cattle slurry and chemical fertilizer on the soil phosphorus balance of spring maize fields in northeast China. *J. Agro-Environ. Sci*. **2022**, *41*, 2232-2240.
37. Xie, L.; Li, L.; Xie, J.; Wang, J.; Zhou, Y.; Chen, Q.; Setorkwami, F. Effects of substitution of chemical fertilizer by organic fertilizer on maize growth and field carbon emission in dry farming area of Longzhong, Gansu Province. *J. Plant Nutr. Fert*. **2022**, *28*, 1029-1038.
38. Tao, S.; He, Z.; Zhang, M.; Chen, Y.; Dai, M.; Zhang, H.; Ding,W. Effects of Organic(Biogas Slurry) Substitution and Reduced Fertilization on Crop Yield and Fertilizer Utilization under Wheat Jade Rotation Mode. *Journal of Sichuan Agricultural University* **2022**, *40*, 714-720.
39. Li, Y.; Harihash Y.; Yu, J.; Li, Q. Application of Chemical Fertilizer Combined with Manure Improves Maize Yield, Nitrogen Absorption and Grain Zinc Content. *Xinjiang Agric. Sci*. **2022**, *59*, 1077-1083.
40. Liu, J.; Li, T.; Zhang, Y.; Wu, H. Analysis of the response and driving factors of corn yield after short-term reduction of chemical fertilizers. *Soil Fert. Sci. China* **2022**, *300*, 66-73.
41. Song, J.; Wang, H.; Zhang, S.; Peng, C.; Lu, C.; Chi, G.; Chen, X. Effects of fertilization and straw return on nutrient distribution along soil profile in black soil croplands. *Chin. J. Ecol.* **2022**, *41*, 108-115.
42. Shi, G.; Dong, H.; Yu, S.; Zhu, G.; Mou, X.; Zheng, Z.; Ren, H. Effects of Different Application Methods of Slow and Controlled Release Fertilizer on Summer Maize Yield, Fertilizer Use Efficiency and Economic Benefits. *Shandong Agric. Sci*. **2021**, *53*, 80-84.
43. Liu, X.; Zheng, C.; Cao, W.; Dang, H.; Cao, C.; Li, X.; Li, K.; Ma, J. Effects of Long-Term Located Fertilization on Soil Organic Matter, Nitrogen Forms and Crop Yields. *Crops* **2021**, *203*, 130-135.
44. Wu, Z.; Ma, H.; Li, R.; Yu, L.; Ma, J.; Guo, L.; Jiang, G. Effects of Ultra-High Organic Fertilizer Application on Maize Growth and Yield in Organic Dry-Land Field of Loess Plateau in the First Year. *Shandong Agric. Sci*. **2021**, *53*, 57-63.
45. Lv, J.; Wang, X.; Li, T.; Kou, C. Ammonia emission characteristics and emission coefficients of wheat and corn rotation cropland under different fertilization methods in lime concretion black soil. *Chin. J. Eco-Agric*. **2020**, *28*, 1869-1879.
46. Cao, H.; Xie, J.; QiangJiu, C.; Guo, L.; Hong, J.; Jing, Y.; Meng, H. Effects of fertilization regimes on carbon and nitrogen contents ofaggregates and maize yield in reclaimed soils. *Trans. Chin. Soc. Agric. Eng.* **2020**, *36*, 135-143.
47. Guo, S.; Cui, Z.; Shi, W.; Cheng, H.; Xiao, R.; Xiao, Z.; Zhao, Y.; Cao, J. Effects of nitrogen,phosphorus,potassium and their combined application on nutrient absorption and utilization of seed maize. *Agric. Res. Arid Areas* **2020**, *38*, 221-226.
48. Wang, G.; Li, Y.; Wang, J. Effects of Different Fertilization Patterns on Growth Characteristic and Nitrogen Use Efficiency of Corn. *Southwest China J.Agric.Sci*. **2019**, *32*, 2119-2125.
49. E, S.; Ding, N.; Li, L.; Yuan, J.; Che, Z.; Zhou, H.; Shang, L. Contribution of Fertilization to Accumulative Crop Yield and Soil Fertility in Heilu Soil Region of the Loess Plateau. *Acta Pedol. Sin*. **2019**, *56*, 195-206.
50. Xin, X.L.; Qin, S.W.; Zhang, J.B.; Zhu, A.; Yang, W.L.; Zhang, X.F. Yield, phosphorus use efficiency and balance response to substituting long-term chemical fertilizer use with organic manure in a wheat-maize system. *Field Crops Res.* **2017**, *208*, 27-33.
51. Xu, F.; Liu, Y.; Du, W.; Li, C.; Xu, M.; Xie, T.; Yin, Y.; Guo, H. Response of soil bacterial communities, antibiotic residuals, and crop yields to organic fertilizer substitution in North China under wheat- maize rotation. *Sci. Total Environ.* **2021**, *785*, 147248.
52. Liang, Y.; Al-Kaisi, M.; Yuan, J.; Liu, J.; Zhang, H.; Wang, L.; Cai, H.; Ren, J. Effect of chemical fertilizer and straw-derived organic amendments on continuous maize yield, soil carbon sequestration and soil quality in a Chinese Mollisol. *Agric., Ecosyst. Environ.* **2021**, *314*, 107403.
53. Zhang, Y.; Li, C.; Wang, Y.; Hu, Y.; Christie, P.; Zhang, J.L.; Li, X. Maize yield and soil fertility with combined use of compost and inorganic fertilizers on a calcareous soil on the North

China Plain. *Soil Tillage Res.* **2016**, *155*, 85-94.
54. Mia, H.T.; Lu, J.L.; Xu, M.G.; Zhang, W.J.; Huang, S.M.; Peng, C.; Chen, L.M. Carbon and nitrogen allocations in corn grown in Central and Northeast China: different responses to fertilization treatments. *J. Integr. Agric.* **2015**, *14*, 1212-1221.
55. Zou, W.; Si, B.; Han, X.; Jiang, H. The effect of long-term fertilization on soil water storage and water deficit in the Black Soil Zone in northeast China. *Can. J. Plant Sci.* **2012**, *92*, 439-448.
56. Chen, Q.; Xie, J.; Li, L.; Wang, L.; Zhou, Y.; Li, J.; Wang, J. Effects of different proportions of organic fertilizer substitutes for chemical fertilizer on growth characteristics and water use efficiency of maize. *Agric. Res. Arid Areas* **2021**, *39*, 162-170.
57. Zhang, D.; Chi, B.; Zhang, W.; Li, H.; Huang, X.; Liu, E.; Fan, X. Influence of Fertilizer Application Levels on Yield and WUE of Dryland Maize in Different Precipitation Years. *Acta Agric. Boreali-Occident. Sin.* **2012**, *21*, 84-90.
58. Zhang, X.; An, J.; Lou, C. Effects of Different Nitrogen Application Methods on Dynamic Changes of Soil Inorganic Nitrogen in Corn Field. *J. Maize Sci.* **2015**, *23*, 143-148.
59. Gao, H.; Zhu, P.; Peng, C.; Zhang, X.; Li, Q.; Zhang, W. Effects of Different Fertilization Methods on Soil Moisture and Temperature Characteristics of Spring Maize Fields in Northeast China. *J. Soil Water Conserv*. **2015**, 29, 195-200.
60. Pang, J.; Wang, Y.; Liu, C.; Gao, Y.; Liu, D.; Zhang, Y.; Yang, B.; Jia, Z.; Zhang, P. Effects of fertilization on soil moisture and maize yield in rainfed farmland with ridge mulching-furrow planting system. *J. Plant Nutr. Fert*. **2021**, *27*, 826-836.
61. Zheng, L.; Wu, S.; Dang, T. Effects of Different Fertilization Modes on Spring Maize Yield, Water Use Efficiency and Nitrate Nitrogen Residue. *J. Soil Water Conserv*. **2019**, *33*, 221-227.
62. Guo, T.; Xie, Y.; Zhang, P.; Liu, X.; Jiang, X. Effects of Different Patterns of Planting and Fertilization on Soil Moisture and Water Use Efficiency of Spring Maize on Dryland. *J. Soil Water Conserv*. **2015**, *29*, 231-238.
63. Zhang, W.; Wang, X.; Zhou, Y. Effects of Straw Mulching and Fertilization on Water and Nitrogen Use Efficiency of Summer Maize. *J. Soil Water Conserv*. **2016**, *30*, 139-146.
64. Wang, X.; Xing, Y.; Li, Z. Effect of Mulching and Nitrogen Fertilizer on Maize Yield,Distribution and Fate of Nitrogen in Root Layer. *Sci. Agric. Sin.* **2016**, *49*, 3944-3957.
65. Wang, H.; Wang, S.; Xu, Z.; Li, J. Effect of tillage and fertilization on water use efficiency of maize in dryland conditions. *Chin. J. Eco-Agric.* **2017**, *25*, 856-864.
66. Wang, X.; Cai, L.; Qi, P.; Wang, Y.; Chen, X.; Wu, J.; Zhang, R. Effects of alternative fertilizer options on soil $CO_2$ emission and carbon pool management index in a dryland soil. *Acta Pratac. Sin.* **2021**, *30*, 32-45.
67. Zou, W.; Han, X.; Wang, S.; Jiang, H.; Yang, C. Water Consumption by Maize Under Different Fertilization Managements in Black Soil Zone of Northeast China. *J. Ecol. Rural Environ.* **2012**, *28*, 681-686.
68. Zhou, H.; Yang, Z.; Li, H.; Guan, C. Influence of fertilization and rainfall distribution on yield and water use efficiency of maize in dryland. *Agric. Res. Arid Areas* **2004**, *3*, 27-31.
69. He, M.; Wang, L.; Wang, Y.; Shen, X.; Zhang, Y.; Zhu, P. Characteristic of black soil respiration and its influencing factors under long-term fertilization regimes. *Trans. Chin. Soc. Agric. Eng.* **2018**, *34*, 151-161.
70. Chen, G.; Wang, P.; Tao, H.; Zhang, Z. Effects of combined application of organic and chemical fertilizers on the yield and water utilization of spring maize in dryland. *Agric. Res. Arid Areas* **2012**, *30*, 139-144.
71. Zhang, X.; Dong, Z.; Wu, X.; Gan, Y.; Chen, X.; Xia, H.; Kamran, M.; Jia, Z.; Han, Q.; Shayakhmetova, A.; et al. Matching fertilization with water availability enhances maize productivity and water use efficiency in a semi-arid area: Mechanisms and solutions. *Soil Tillage Res.* **2021**, 105164.
72. Wang, X.; Nie, J.; Wang, P.; Zhao, J.; Yang, Y.; Wang, S.; Zeng, Z.; Zang, H. Does the replacement of chemical fertilizer nitrogen by manure bene fi t water use e ffi ciency of winter wheat - summer maize systems? *Agric. Water Manage.* **2021**, *243*, 106428.

73. Peng, Z.; Wang, L.; Xie, J.; Li, L.; Coulter, J.A.; Zhang, R.; Luo, Z.; Cai, L.; Carberry, P.; Whitbread, A. Conservation tillage increases yield and precipitation use efficiency of wheat on the semi-arid Loess Plateau of China. *Agric. Water Manage.* **2020**, *231*, 106024.
74. Hu, S.; Wang, X. Effects of Fertilization on Biological Properties and Water and Fertilizer Use Efficiency of Maize in Black Soil Region of Northeast China. *J. Soil Water Conserv.* **2017**, *31*, 219-226.
75. Shu, F.; Dong, Q.; Feng, H.;Li, C.; Han, J.; Zhang, T. Effects of Different Organic Materials on Water Movement in Gully Land Consolidation Soil on the Loess Plateau. *J. Soil Water Conserv.* **2022**, *36*, 74-79.
76. Zhang, M. Effects of Water and Fertilizer Integration on Maize Yield and Water-Nitrogen Use Efficiency under Micro-Sprinkler Irrigation. *Shandong Agricultural University* **2022**, 17.
